# Modification of Barley Plant Productivity Through Regulation of Cytokinin Content by Reverse-Genetics Approaches

**DOI:** 10.3389/fpls.2018.01676

**Published:** 2018-11-27

**Authors:** Katarína Holubová, Goetz Hensel, Petr Vojta, Petr Tarkowski, Véronique Bergougnoux, Petr Galuszka

**Affiliations:** ^1^Department of Molecular Biology, Centre of the Region Haná for Biotechnological and Agricultural Research, Faculty of Science, Palacký University, Olomouc, Czechia; ^2^Plant Reproductive Biology, Leibniz Institute of Plant Genetics and Crop Plant Research (IPK) Gatersleben, Gatersleben, Germany; ^3^Institute of Molecular and Translation Medicine, Faculty of Medicine and Dentistry, Palacký University, Olomouc, Czechia; ^4^Central Laboratories and Research Support, Centre of the Region Haná for Biotechnological and Agricultural Research, Faculty of Science, Palacký University, Olomouc, Czechia; ^5^Department of Genetic Resources for Vegetables, Medicinal and Special Plants, Crop Research Institute, Centre of the Region Haná for Biotechnological and Agricultural Research, Olomouc, Czechia

**Keywords:** cytokinin, barley, CRISPR-Cas9, silencing, yield

## Abstract

Barley is one of the most important cereals, which is used for breweries, animal and human feeds. Genetic manipulation of plant hormone cytokinins may influence several physiological processes, besides others stress tolerance, root formation and crop yield. *In planta*, endogenous cytokinin status is finely regulated by the enzyme cytokinin dehydrogenase (EC 1.5.99.12; CKX), that irreversible degrades the side chain of adenine-derived isoprenoid cytokinins. Increasing grain yield by mean of manipulation of endogenous cytokinin content was assayed by the silencing of the *HvCKX1* gene. Moreover, to elucidate the putative role of *HvCKX1* gene on grain production, knocked-out *Hvckx1* mutant plants were generated using the RNA-guided Cas9 system. Homozygote transgenic plants with silenced *HvCKX1* gene and azygous knock-out *Hvckx1* mutants have been selected and analyzed. Both reduced expression of *HvCKX1* gene and CKX activity were measured in different stages of barley grain development. Phenotyping of the transgenic lines revealed reduced root growth, however, plants produced more tillers and grains than azygous wild-type controls and the total yield was increased up to 15 per cent. Although plant productivity was increased, total grain biomass was decreased to 80% of WT grains. RNA-seq analysis of knock-down transgenic lines revealed that several important macronutrient transporters were downregulated in the stage of massive starch accumulation. It suggests that local accumulation of cytokinins negatively affected nutrients flow resulting in reduced grain biomass. Obtained results confirmed the key role of HvCKX1 for regulation of cytokinin content in barley.

## Introduction

The small grain cereal model crop barley belongs to the most important cereals grown worldwide. In terms of acreage, it ranges on ranks fourth in cereal production according to FAO (FAOSTAT). Due to its high adaptability, it can be grown on different soil types and altitudes. In addition, the genomic sequence is available (Mascher et al., 2017) and many tools including efficient genetic transformation were developed (Hensel et al., 2009). Amongst adaptation to different biotic and abiotic factors, the increase in crops productivity is one of the main goals in agriculture. One very promising tool to improve the plant productivity is the manipulation of cytokinin (CK) metabolism. The CKs are naturally occurring hormones, which regulate plant morphogenesis, flowering, seed development and germination as well as nutrient uptake into sink organs (Kieber and Schaller, 2018). Structurally they are either isoprenoid or aromatic CKs, while the former is found more frequently in plants (Sakakibara, 2006). Depending on their side-chains they are further divided into *N^6^*-(Δ^2^-isopentenyl)-adenine (iP), *trans*-zeatin (*t*Z), *cis*-zeatin (*c*Z), and dihydrozeatin (DHZ). For instance, *t*Z- and iP-type CKs are the major forms in Arabidopsis, whereas substantial amounts of *c*Z-type CKs are found in maize, rice, and chickpea (Sakakibara, 2006). Genetic engineering of CK metabolism in model plants reveals enormous potential in agricultural applications such as remodeling plant architecture, increasing stress tolerance and enhancing crop yield (Zalabák et al., 2013).

One of the possibilities how to modify the CK content is through regulation of *IPT* gene, coding for isopentenyl transferase, which catalyzed the rate-limiting step in CK biosynthesis. This approach was first used in tobacco plants, where the *IPT* gene expression was driven by a highly specific senescence promoter (*Senescence associated-gene 2*, *SAG2*) (Gan and Amasino, 1995). These transgenic tobacco plants exhibited prolonged photosynthetic life-span resulting in 50% increase of both dry weights of aerial part and seed yield in comparison to wild-type plants (Gan and Amasino, 1995; Jordi et al., 2000). The *IPT*-based technology was transferred also to agronomically important crop plants. Despite/whereas the inhibition of senescence due to *IPT* overexpression was observed in all transgenic plants transformed with the *SAG12-IPT* construct, the impact on yield varied between individual crops (Gregersen et al., 2013). For example, in *SAG12-IPT* transgenic wheat, the delayed senescence had no positive effect on yield parameters (Sýkorová et al., 2008). In opposite, *SAG12-IPT* transgenic rice plants with such a delayed senescence produced a higher number of seeds and panicles; nevertheless, no effect on total grain yield was observed as grains were less filled (Lin et al., 2002; Liu et al., 2010). In summary, a long vegetative period is definitely favorable for higher biomass production but it may be noxious for grain yield, as the reproductive phase is shortened or onset of nutrient mobilization from source to sink is shifted (Gregersen et al., 2013).

Another approach how to increase crop productivity through the manipulation of CK homeostasis employed the *CKX* gene, coding for cytokinin dehydrogenase, which catalyzes the irreversible degradation of CKs and is thus a key regulator of the CK content in the plants. In the previous study on Habataki variety of rice, a crucial role of OsCKX2 in the formation and control of inflorescence meristem activity was shown. Indeed, the loss-of-function mutation of this gene led to a significant increase in grain number (Ashikari et al., 2005). The fundamental role of CKX enzyme in the formation of inflorescence meristem was confirmed recently in Arabidopsis, where the double *ckx* knock-out led to 55% yield improvement by an increased seed set per silique (Bartrina et al., 2011). Similarly, RNAi-silencing of *HvCKX1* gene, coding for the CKX enzyme mostly present in barley aleuronic layer surrounding the endosperm resulted in higher grain yield (Zalewski et al., 2010). All these studies underline the crucial role of CKs in developmental processes and give the example of how the manipulation of a single gene can positively influence the plant productivity.

The development of targeted mutagenesis based on the Cas9 protein associated with Type II Clustered Regulatory Interspaced Short Palindromic Repeats (CRISPR) enabled the genome editing at the specific target site. CRISPR-Cas9 system uses a small non-coding RNA, known as single guide RNA (sgRNA) to direct Cas9 nuclease to the DNA target where it generates double-strand breaks (DSB). These breaks are subsequently repaired by cells endogenous DNA repair mechanisms, the non-homologous end-joining (NHEJ) or the homology-directed repair. The preferential NHEJ is an error-prone process resulting in imprecise ligation of double-strand breaks and thus the creation of nucleotide insertion/deletions at the site of interest (Voytas, 2013; Puchta and Fauser, 2014). This system offers a flexible, easy and relatively cheap method for genome editing, which was recently successfully used in the modification of barley genome (Lawrenson et al., 2015; Watanabe et al., 2016; Holme et al., 2017). In addition, induced mutations were stably inherited in progeny plants.

In our approach, we used the RNAi-based silencing technique and RNA-guided Cas9 system to generate transgenic barley plants with silenced or knocked-out *HvCKX1* gene. On several independent homozygous lines, we described how the *HvCKX1* silencing and knock-out influenced the development and yield of barley plants.

## Materials and Methods

### Preparation of Constructs for Silencing *HvCKX1* Gene

The construct designed for silencing of *HvCKX1* (HORVU3Hr1G019850) gene (KD-HvCKX1) consists of the 5′ end of the ORF and 3′ untranslated region (3′UTR) of *HvCKX1* gene (250 bp; Supplementary Figure S1). The 3′UTR region was used to avoid the silencing of orthologous non-targeted *HvCKX* genes. The *SC_HvCKX1* fragment was synthesized and subcloned into pMA vector by GeneArt (Thermo Fischer Scientific, Waltham, United States). Subsequently, it was subcloned into the Gateway entry vector pENTR1A at restriction sites *Bam*HI and *Eco*RV. Two copies of *SC_HvCKX1* were inserted in the opposite orientation into the vector pBract207 downstream of the maize *UBIQUITIN 1* promoter (provided by John Innes Centre, Norwich, United Kingdom^1^) via Gateway LR recombination reaction (Invitrogen, Carlsbad, CA, United States). The final vector was Sanger sequenced (SeqMe, Czechia) using three primers (Supplementary Table S1) to confirm the right orientation of both *SC_HvCKX1* copies, and electrotransformed into *Agrobacterium tumefaciens* (*A. tum.*) strain AGL1 together with the helper vector pSoup. *A. tum.* containing pBract207::*SC_HvCKX1* was mixed with glycerol in ratio 1:1 (50% solution, v/v) and stored at -80°C.

### Preparation of *HvCKX1* Knock-Out Construct

The generation of the *HvCKX1*-specific Cas9 construct was essentially done as described previously (Budhagatapalli et al., 2016). The target sequence for the guide RNA has been selected within the first exon of *HvCKX1* gene using DESKGEN KNOCKIN tool^2^. For easy screening of the transformants, the guide RNA sequence contains a *Bsa*HI restriction site, close to the protospacer adjacent motif (PAM) motive. Gene-specific oligos (Supplementary Table S2) were annealed and integrated into the *Bsa*I-digested pSH91 vector. The guide RNA-Cas9 expression cassette was sequenced by Sanger sequencing and subcloned into the p6i-d35S-TE9 vector (DNA-Cloning-Service, Hamburg, Germany) using *Sfi*I restriction enzyme giving rise to plasmid pGH437 (KO-CKX1). Transfer into *A. tum.* strain AGL1 was performed as described above.

### Preparation of *HvCKX1* Target Construct and Transient Expression Tests

In order to test the activity of the KO-CKX1 construct, transient expression assays in barley leaves were conducted according to Budhagatapalli et al. (2016). For this purpose, the guide RNA target site of *HvCKX1* together with its PAM sequence (annealed oligos; Supplementary Table S2) was integrated into *Eco*RI/*Bam*HI-sites of the pNB1 vector using standard cloning procedures, generating the plasmid pGH314. After Sanger sequencing, the pGH314 was used for transient expression tests via particle bombardment of barley leaves (Budhagatapalli et al., 2016). Leaf explants of 6–7 days old seedlings were transiently transformed using a PDS-1000/He Hepta^TM^ device equipped with an 1100 psi rupture disk (Bio-Rad, Munich, Germany). Each set of explants were co-bombarded twice using pGH314, pGH437 (KO-CKX1) and pNB2 (constitutive *mCherry* for normalization) vectors (Budhagatapalli et al., 2016) with a total amount of 16–19 μg plasmid DNA, then incubated at room temperature for 1 day before assaying for fluorescence.

The frequency of KO-CKX1 construct induced mutation was determined from the ratio between the number of yellow- (YFP) and red-fluorescent (mCherry) cells. For this, a total of six barley leaves were co-bombarded with the respective constructs and analyzed with a fluorescent microscope [Zeiss LSM780 confocal laser microscope (Carl Zeiss, Jena, Germany)]. YFP fluorescence was visualized using 514 nm laser line in combination with 517–560 nm bandpass; mCherry fluorescence was visualized with a 561 nm laser line in combination with 570–620 nm bandpass. The experiment was repeated twice.

### Plant Material and Barley Transformation Procedures

Plants of the spring barley cultivar Golden Promise (wild-type; WT), were grown in phytotron with the photoperiod of 15°C/16 h/light and 12°C/8 h/dark. The light source was a combination of mercury tungsten lamps and sodium lamps providing the intensity of 500 μmol m^-2^ s^-1^ at the level of plant tops. Plants were cultivated in a 2:1 mixture of soil and perlite (Perlit Ltd., Czechia) and were fertilized every 14 days with YaraMila Complex (AgroCS, Czechia). Transgenic barley plants with the silenced *HvCKX1* gene (KD-CKX1) were prepared from immature embryos according to Harwood et al. (2009). *Hvckx1* mutant plants (KO-CKX1) were generated at IPK Gatersleben as published previously (Hensel et al., 2008). All transformants were selected on 50 mg L^-1^ hygromycin.

### Screening of Transformants With Silenced *HvCKX1* Gene

T0, T1, and T2 generation of transgenic plants were screened for the presence of both copies of *SC_HvCKX1* (sense and antisense). The leaves of transgenic barley plants were harvested and used for the isolation of genomic DNA (gDNA) (Edwards et al., 1991). Sense and antisense *SC_HvCKX1* was amplified using GoTaq polymerase (Promega, Madison, United States) with specific primers and PCR conditions (Supplementary Tables S1, S4). The PCR products were assessed by agarose gel electrophoresis and compared with 1 kb Plus DNA marker (Thermo Fischer Scientific, Waltham, MA, United States). Products of a 298 and 477 bp indicated the presence of sense and antisense component of *SC_HvCKX1*.

T0 transgenic plants, positive for the presence of both *SC_HvCKX1* copies, were harvested and all grains from one to two spikes (around 30 grains) were put to the soil to produce T1-plants, which were subsequently screened for the presence of the *SC_HvCKX1.* The transgenic progeny with 3:1 segregation (presence/absence of transgene) were grown to maturity; 15 to 20 T2-embryos from one spike of each plant were isolated and incubated 14 days on the regeneration medium with hygromycin for selection (50 mg L^-1^) in the environmental chamber with the photoperiod of 24°C/16 h/light and 22°C/8 h/dark (160 μmol m^-2^ s^-1^). As a negative control, embryos isolated from WT barley plants were incubated under the same conditions. The T1 transgenic plants, giving 100% regeneration from T2-embryos under the selection pressure were identified as putative homozygotes. For confirmation, all T2 plants are grown from one spike of putative homozygotes where screened for the presence of both copies of *SC_HvCKX1* to confirm the zygosity.

T2 seedlings of all selected transgenic lines were submitted for copy number analysis by iDNA Genetics service (Norwich, United Kingdom^3^).

### Screening of *Hvckx1* Mutant Plants

Genomic DNA was prepared from young seedling (Pallotta et al., 2000). Between 50 and 100 ng of isolated gDNA was used into 20 μL PCR reaction. Products were amplified by GoTaq polymerase with sets of primers detailed in Supplementary Table S2 to test the presence of binary vector and PCR conditions (Supplementary Table S4). The reaction products were purified using PCR purification kit (Machery-Nagel, Düren, Germany) used for amplicon restriction analysis (PCR/RE), visualized on 1% agarose gel containing ethidium bromide and sequenced.

Primary transformants containing the KO-CKX1 construct were tested for possible mutations on the target site. The 756 bp PCR product, covering part of the first *HvCKX1* exon, was digested with *Bsa*HI (Thermo Fischer Scientific, Waltham, MA, United States). Amplicons with mutations on the target site gave the restriction digest products of 582, 114, and 59 bp and non-mutated amplicons products of 355, 228, 114, and 59 bp. PCR products, positive for the mutation, were cloned into a pGEM-T vector (Promega, Madison, United States). After blue-white selection, plasmid DNA was isolated from 10 positive clones and Sanger sequenced. Alternatively, PCR products were sequenced directly, excluding the cloning step (in a subsequent generation).

T1 and T2 generation of *Hvckx1* mutant plants were screened by the same procedure described above. Azygous plants containing the same mutation in both alleles, but did not contain the KO-CKX1 T-DNA were identified as homozygote lines and used for further analysis.

### Assessment of DNA Ploidy

DNA ploidy levels were inferred from the relative fluorescence intensities of PI-stained nuclei using flow cytometry (Shapiro, 2003). Small pieces of leaf tissue were chopped in a Petri dish containing 500 μL of Otto I buffer (0.1 M citric acid, 0.5% Tween 20). The crude suspension of isolated nuclei was filtered through a 50-μm nylon mesh. 1 ml of Otto II buffer (0.4 M Na_2_HPO_4_), supplemented with 50 μg mL^-1^ RNase and 50 μg mL^-1^ propidium iodide, was added. Relative fluorescence intensity of at least 3,000 nuclei was recorded using Partec PAS flow cytometer (Partec GmbH, Münster, Germany) equipped with a high-pressure mercury arc lamp. Data were analyzed using the FlowMax software (Partec, GmbH, Münster, Germany). The gain of the instrument was adjusted the way that the peak representing control plant G0/G1 nuclei appeared on channel 100.

### qPCR Analysis

Plant material collected from 1-week old roots and spikes of BBCH stages 49 [booting: first awns visible (in awned forms only)], 59 (end of heading: inflorescence fully emerged), 71 (watery ripe: first grains have reached half their final size), and 75 (medium milk: grain content milky, grains reached final size, still green), grown in phytotron under the conditions described above, were harvested, frozen in liquid nitrogen and lyophilized 2–3 days by ScanVac Freeze Dryer (Labogene, Denmark). The lyophilized material was homogenized by vibration mill. Total RNA was isolated by RNAqueous^®^ Total RNA Isolation kit (samples from roots) according to supplier’s manual (Thermo Fischer Scientific, Waltham, MA, United States). Alternatively, RNA was isolated using TRI-reagent (samples from spikes; Thermo Fischer Scientific, Waltham, MA, United States). cDNA was obtained from 2 μg of total RNA using a *RevertAid* First Strand cDNA Synthesis Kit (Thermo Fischer Scientific, Waltham, MA, United States), then amplified by qPCR in 5/10 μL reaction mixtures containing TaqMan Gene Expression Master Mix or Power SYBR Green PCR Master Mix (Thermo Fischer Scientific, Waltham, MA, United States), 300 nM of each primer and, when the TaqMan chemistry was used, 250 nM of specific 5′end 6-carboxyfluorescein (FAM) dye and 3′end 5(6)-carboxytetramethylrhodamine (TAMRA) quencher. Reactions were run in a StepOnePlus^TM^ or ViiA7 Real-Time PCR Systems using the default program (Thermo Fischer Scientific, Waltham, MA, United States). For 5 μL reaction, pipetting was performed automatically by pipetting robot Agilent Bravo 04318-201 (Agilent Technologies, Santa Clara, CA, United States). Primers and TaqMan probes were designed using Primer Express 3.0 software (Thermo Fischer Scientific, Waltham, MA, United States; Supplementary Table S3). The most consistently expressed genes in targeted tissue were selected by Genevestigator software (Hruz et al., 2008) and used as reference genes (*Actin* – HORVU2Hr1G004460; *Elongation factor 2* – HORVU5Hr1G116580.8; *ATP-binding gene* – HORVU3Hr1G022710; *Nucleic acid binding gene* – HORVU1Hr1G061690.2). Five to eight biological and two technical replicates were measured per each transgenic barley line. Relative gene expression was determined by the ΔΔ*C*t method.

### RNA-Seq Analysis

Barley spikes of BBCH stages 49 (booting) and 59 (heading) from the plants grown in the field were used as samples. Three μg of total RNA from each sample, extracted with RNAqueous kit (Thermo Fischer Scientific, Waltham, MA, United States) and treated with TURBO DNA-free kit (Thermo Fischer Scientific, Waltham, MA, United States) was used for cDNA library preparation by Illumina^®^ TruSeq^®^ Stranded mRNA Sample Preparation Kit (Illumina, Madison, WI, United States). Library concentration was assessed with a Kapa Library Quantification Kit (Roche, Basel, Switzerland) and all libraries were pooled to a final 8 pM concentration for cluster generation and sequencing. The clusters were generated using an Illumina^®^ TruSeq^®^ SR Cluster Kit v3 cBot HS and sequenced on HiSeq SR Flow Cell v3 with a HiSeq 2500 Sequencing System. Three independent libraries were prepared for each genotype in each time-point (3 pooled spikes in each).

The reads generated by sequencing were mapped to the reference genome of Hordeum_082214v1.30 (Ensembl Plants) using the STAR_2.4.2a (Dobin et al., 2013) with default parameters. Reads quantification was processed by featureCounts, subread-1.5.2 (Liao et al., 2014) against GTF/GFF file Hordeum_vulgare.082214v1.30.gtf (Ensembl Plants) with respect to the stranded library. The tests for differential gene expression were performed using the DESeq2 package (Love et al., 2014) implemented in R (R Development Core Team, 2008). The classification of differentially expressed genes was performed online by MapMan Mercator tool (Lohse et al., 2014).

### CKX Enzyme Activity Assay

To measure CKX activity, proteins were extracted from homogenized lyophilized plant material (grown in phytotron), collected from 1-week old roots and spikes of BBCH stages 49 (booting), 59 (heading), 71 (watery-ripe), and 75 (medium milk), with 0.2 M Tris/HCl buffer (pH 8.0) containing 150 mM NaCl, 1 mM phenylmethylsulfonyl fluoride, 0.1% Triton X-100 (v/v) and leupeptin (10 μM). Cell debris was removed by centrifugation at 21,000 × *g* for 10 min at 4°C. The protein content was estimated following the Bradford method with bovine serum albumin as a standard (Bradford, 1976). The CKX activity was determined by spectrophotometric assays by the end-point method, where the absorbance of Schiff base of aldehyde originated from cytokinin side chain and *p*-aminophenol is determined at 352 nm (Frébort et al., 2002). Between 200 and 500 μg of total proteins were incubated for 2–8 h in 0.5 mL reaction mixtures containing 100 mM McIlvaine buffer (pH 7.5), 0.1 mM N6-isopentenyladenine (iP) as a substrate and 0.6 mM 2,6-dichlorophenolindophenol as an electron acceptor at 37°C. Five to eight biological and two technical replicates were measured per each transgenic barley line.

### Cytokinin Detection

Spikes of plants grown in phytotron collected in BBCH stages 49 (booting), 59 (heading), and 71 (watery-ripe) and plants grown in the field, collected in stages 49 and 59, were immediately frozen in liquid nitrogen and lyophilized. Lyophilized material (25 mg) was ground by vibration mill and extracted overnight with 1.5 mL of Bieleski solvent at -20°C, followed by an additional re-extraction in 0.5 mL of the same solvent for 1 h at -20°C (Bieleski, 1964). Stable isotope-labeled cytokinin internal standards were added before extraction. Cytokinins were purified using SCX SPE columns and quantified by liquid-chromatography-positive-electrospray-tandem mass spectrometry in multiple reaction monitoring modes (Novák et al., 2008). UHPLC-MS/MS experiments were performed using NEXERA X2 liquid chromatograph coupled with MS-8050 (Shimadzu, Japan) via an electrospray interface. Chromatographic separation was performed using reversed-phase analytical column (BEH C18, 2.1 mm × 50 mm, 1.7 mm; Waters), column thermostat was set at 40°C. Solvent A was 15 mM formic acid adjusted to pH 4.0 by ammonium hydroxide. Solvent B was methanol. Gradient elution was performed at a flow rate of 0.5 mL min^-1^: 0 min, 10% B; 0–8 min linear gradient to 50% B followed by 5 min equilibration to initial conditions. The mass spectrometer settings were as follows: mode ESI+, capillary voltage 0.6 kV, source temperature 400°C, desolvation temperature 250°C, desolvation gas flow 3 L min^-1^. Five to eight biological replicates were measured per each transgenic barley line.

### Hydroponic Experiment

The grains were germinated and grown for 3 days on wet paper tissues in the phytotron with a photoperiod of 24°C/16 h/light and 22°C/8 h/dark. Developed plantlets were put into a piece of foam and dipped in a pot containing Hoagland’s solution (Hoagland and Arnon, 1950). The solution was replaced every 2–3 days. The plants were cultivated for 4 weeks in the environmental chamber with a photoperiod of 15°C/16 h/light and 12°C/8 h/dark and the shoots and roots harvested in three-time points (7, 14, and 28 days after germination). The roots were stored in 30% ethanol and analyzed by Epson Perfection V700 Photo Scanner. Total length and area of roots were determined using WinRHIZO software (Régent Instruments, Québec, Canada). Subsequently, the roots and shoots of analyzed plants were dried 24 h on 50°C and weighed. Nine biological replicates for each transgenic barley line were analyzed at each time point.

### Yield Experiments

For greenhouse experiments conducted in the years 2014 and 2015, 25 of transgenic or control plants were grown in the greenhouse with a photoperiod of 24°C/16 h/light and 22°C/8 h/dark. Plants were cultivated in a 2:1 mixture of soil and perlite (Perlit Ltd., Czechia) and were fertilized every 14 days with YaraMila Complex (AgroCS, Czechia). The mature plants were harvested and the following yield parameters were determined: number of spikes per plant, number of grain per plant, the weight of grains per plant and thousand grain weight (TGW). The collected spikes from each plant were threshed on a threshing machine and the number of grains calculated on the automatic counter.

The field trials were conducted on area belonging to Palacký University in Olomouc from 2016 to 2017. Plants were grown with a density of 200 plants m^-2^ in plots 1.2 m × 21 m (each of transgenic lines in one plot). The weight of grains per square meter was calculated according to the formula: 0.4 (0.2) × TGW × germination rate/100. The planting was performed by tractor and mature plants were harvested by a combine harvester. The plants were assessed for the following parameters: number of spikes per plant (calculated before harvesting, 50 plants per each line), total yield per square meter and TGW.

### Statistics Analysis

All analyses were performed with Statistica v.13.3 (TIBCO Software Inc.). Normality of the data was established by the Shapiro–Wilk test. The non-parametric Kruskal–Wallis ANOVA, followed by multiple comparisons of mean ranks was used to compare the differences between groups.

## Results

### Transgenic Barley Plants With Silenced *HvCKX1* Gene

**FIGURE 1:**
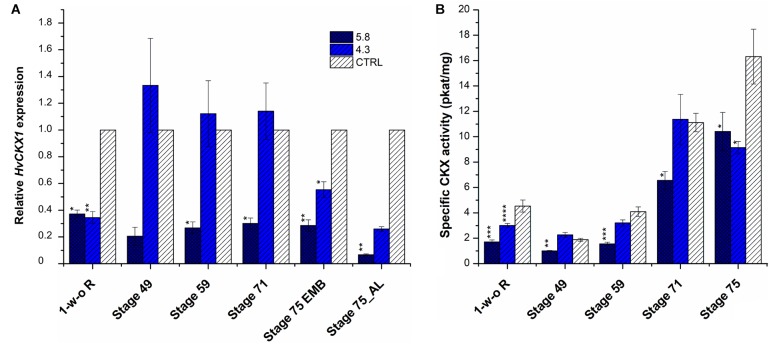
Relative *HvCKX1* expression **(A)** and specific CKX activity (pkat/mg; **B**) in the roots and spikes of KD-CKX1 lines at different stages of development. Expression and activity were measured in 1 week-old roots (1-w-o-R) and four developmental stages of KD-CKX1 lines (5.8 and 4.3) and control plants (CTRL). Developmental stages are named according BBCH scale: stage 49 [booting: first awns visible (in awned forms only)]; stage 59 (end of heading: inflorescence fully emerged); stage 71 (watery ripe: first grains have reached half their final size); stage 75 (medium milk: grain content milky, grains reached final size, still green); EMB, embryo; AL, aleuronic layer. Values are mean ± SE (*n* = 5). A non-parametric Kruskal–Wallis Anova & Median test analysis was performed followed by a *post hoc* multiple comparison of mean rank (Statistica v.13.3); significantly different from the control at ^∗^*p* < 0.01, ^∗∗^*p* < 0.001, ^∗∗∗^*p* < 0.0001, ^∗∗∗∗^*p* < 0.00001. For **(A)**, normalization was done in relation to two reference genes: *Actin* (HORVU2Hr1G004460) and *Elongation factor 2* (HORVU5Hr1G116580.8). Results were expressed as fold change relative to the expression of *HvCKX1* gene in the CTRL grown in the same conditions.

The KD-CKX1 construct was designed to efficiently target the variable 3′end of the *HvCKX1* transcript sequence to exclude silencing of any other member of the barley CKX family (Supplementary Figure S2). A total of 22 primary transgenic plants were generated by *A. tum.*-mediated transformation of barley immature embryos. While 18 plants successfully integrated both inverted repeats, two plants contain only one repeat and two plants contain none of the fragments (Supplementary Figure S3). The estimation of ploidy revealed amongst the 18 positive tested plants, 6 were tetraploid and consequently excluded from further analysis. Out of twelve progeny, eight families segregated in a 3:1 (presence/absence of T-DNA) Mendelian fashion. After transgene confirmation in T2 generation, four homozygous transgenic lines were selected (marked as 5.8, 4.3, 17.10, and 21.4). Two lines (5.8, 4.3) containing one transgene copy in their genome were evaluated for *HvCKX1* transcript level and specific CKX enzymatic activity in four developmental stages of barley grains, BBCH scale 49 [booting: first awns visible (in awned forms only)], 59 (end of heading: inflorescence fully emerged), 71 (Watery ripe: first grains have reached half their final size), 75 (Medium milk: grain content milky, grains reached final size, still green) (Enz and Dachler, 1997) and in 1-week-old roots. As a control, progeny of transgenic line, from which transgene was segregated out in T1 generation, were used (named as azygous control). Reduction of *HvCKX1* expression as well as CKX enzymatic activity was confirmed in both transgenic lines in stage 75 (Figure 1). In stages 59 and 71, *HvCKX1* expression and enzymatic activity was significantly decreased only in line KD-CKX1 5.8 (Figure 1). As the level of silencing and CKX activity varied between the two tested lines, two other homozygous transgenic lines, marked as 17.10 (two transgene copies) and 21.4 (one transgene copy) were added for further analysis.

The expression of the most abundant *HvCKX* and *HvIPT* genes (coding for proteins which regulate the synthesis of cytokinins) were determined as well (genes were selected according to Mrízová et al., 2013). No significant changes in the expression could be observed between homozygous transgenic plants and control plants neither in *HvCKX* genes nor *IPT* genes (Supplementary Table S5). These results confirmed that only *HvCKX1* gene was targeted and silenced.

### Generation of *Hvckx1* Mutant Barley Plants

The target sequence used to guide Cas9 endonuclease was selected at the beginning of the first *HvCKX1* exon. None of the known *HvCKX* genes can be targeted by the selected guide RNA because of high diversity in this region (nucleotides 40–60 of *HvCKX1* gene). Blast search against the barley genome gave 20 putative hits with homologies of 14 to 18 nucleotides of the guide^4^. Detailed sequence analysis revealed that none of the 20 putative off-targets could be targeted by the guide RNA/Cas9 complex because of the lack of the NGG PAM site in close vicinity to the binding motif (Supplementary Figure S4). The functionality of the prepared KO-CKX1 construct was validated using a transient expression assay (Budhagatapalli et al., 2016). Co-bombardment of barley leaves with *HvCKX1*-specific Cas9 endonuclease and pNB1-*HvCKX1* target construct containing PAM led to relative cleavage activity of 92.8% expressed as the ratio between YFP/mCherry positive cells (Table 1).

**Table 1:** Relative cleavage activity of KO-CKX1 construct in transiently transformed barley epidermal cells.

Target gene	Constructs used	Experiment	YFP cells	mCherry cells	Ratio YFP/mCherry cells (%)
*HvCKX1*	pTARGET-*ckx1*	1	11	11	100.0
	+KO-CKX1	2	24	28	85.7
		**Average**	**18 ± 9**	**20 ± 11**	**92.8**

**FIGURE 2:**
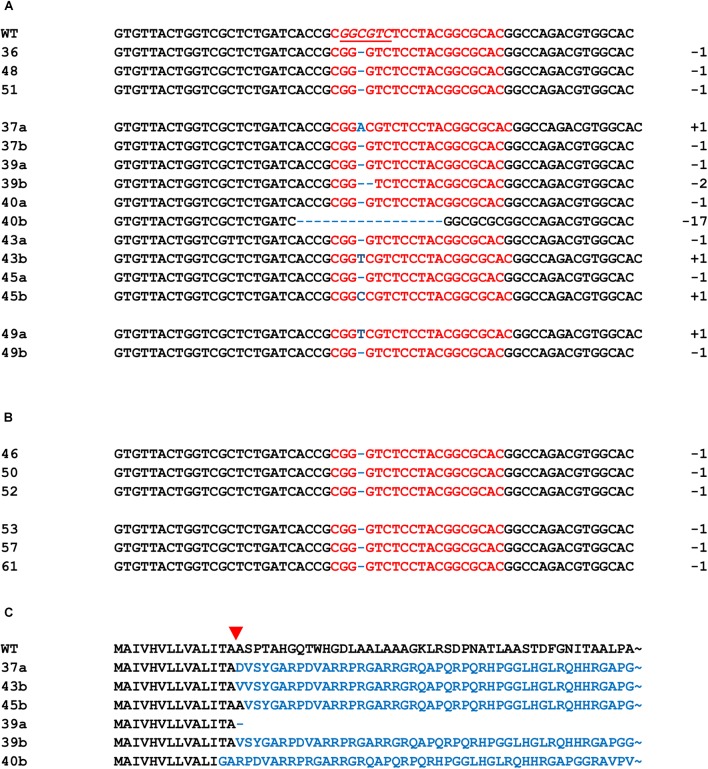
Modification of HvCKX1 sequence induced by the KO-CKX1 construct in primary transgenic mutant plants. *HvCKX1* sequence alignment of the nine mutants, where the mutation occurred in both alleles **(A)**; six mutants, where the mutation occurred in one allele **(B)** and WT *HvCKX1* sequence. Deduced amino acid sequences of the mutant proteins **(C)**. The red sequence represents the Cas9 target site and underlined italics sequence represents *Bsa*HI restriction site. The number of nucleotides deleted (dashed) or inserted (blue color) is shown to the right side of each sequence. The names of the lines are shown to left site of each sequence, where (a) and (b) of the lines 37, 39, 40, 43, 45, 49 represent two different sequences occurred. The red triangle indicates the DSB induction site.

Subsequently, 29 primary transformants were generated by *A. tum.*-mediated transformation of barley immature embryos. Out of 29 transgenic plants, 6 were tetraploids and therefore excluded from further analysis. Resulting 23 primary transformants were evaluated for the presence of mutations in the target sequence based on PCR/RE analysis with *Bsa*HI restriction enzyme. A mutation was revealed in 15 out of 23 primary transformants (Supplementary Figure S5). Sequencing of the target region confirmed the genetic modification in all 15 transgenic plants (Figure 2). Mutations were detected 3 nucleotides upstream of PAM sequence. In 9 out of 15 mutated lines, genetic modification occurred in both alleles because none of the 10 analyzed subclones contained WT or any other sequence (Figure 2A). The loss of *Hvckx1* function was caused by deletions ranging in size from 1 to 17 bp or by 1 bp insertion (Figure 2), while the deletion of 1 bp was found in all other plants. Mutations induced frame shifts, leading to the presence of a premature stop codon or the production of non-sense protein (Figure 2C). Overall, 6 independent mutations occurred in 15 transgenic events. Four homozygous *Hvckx1* mutant plants (named as 37.8, 39.4, 40.4, and 48.1) were selected based on sequence analysis in T1 transformants and used for further evaluation. Prior to sequence analysis, T1 transformants were validated for the presence of T-DNA and those which lost the transgene by segregation were used for further sequencing analysis. Since the induced mutation does not interfere with the expression of the *Hvckx1* gene, the expression level was not measured in the mutant plants.

### CKX Activity During Grain Development

In KD-CKX1 lines CKX activity was estimated in two independent experiments. In the first experiment, spikes of BBCH stages 49 (booting), 59 (heading), 71 (watery ripe), and 75 (medium milk) (Enz and Dachler, 1997) and 1-week-old roots of two KD-CKX1 lines 5.8 and 4.3 were evaluated (Figure 1B). KD-CKX1 line 5.8 had significantly decreased CKX activity (to 20–60%) in all tested samples in comparison with azygous control. However, in KD-CKX1 line 4.3 CKX activity significantly declined just in 1-week-old roots (to 66% of control activity), but was comparable to the activity of azygous control in spikes of all tested developmental stages (Figure 1B).

**FIGURE 3:**
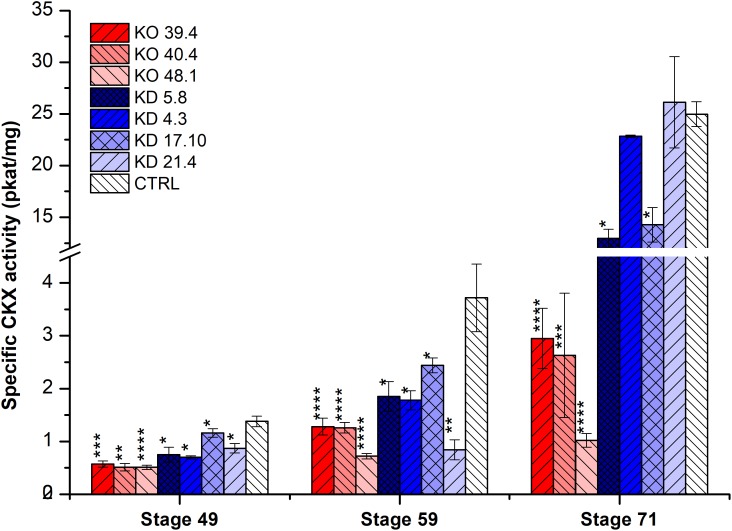
Specific enzymatic CKX activity (pkat/mg) in the spike of KD-CKX1 and KO-CKX1 transgenic lines at different stages of development. Activity was measured at three developmental stages of KD-CKX1 lines (5.8, 4.3, 17.10, and 21.4), KO-CKX1 mutant lines (39.4, 40.4, and 48.1) and control plants. Stages are named according BBCH scale: stage 49 [booting: first awns visible (in awned forms only)]; stage 59 (end of heading: inflorescence fully emerged); stage 71 (watery ripe: first grains have reached half their final size). Values are mean ± SE (*n* = 5 to 8). A non-parametric Kruskal–Wallis Anova & Median test analysis was performed followed by a *post hoc* multiple comparison of mean rank (Statistica v. 13.3); ^∗^*p* < 0.01, ^∗∗^*p* < 0.001, ^∗∗∗^*p* < 0.0001, ^∗∗∗∗^*p* < 0.00001.

In the second experiment, three developmental stages (49, 59, and 71) were estimated in four KD-CKX1 (5.8, 4.3, 17.10, and 21.4), and three KO-CKX1 mutant lines (48.1, 40.4, and 39.4) as well as azygous segregants (Figure 3). CKX activity was significantly decreased in KD-CKX1 lines 5.8 and 17.10 and in all KO-CKX1 mutant lines in three stages tested. KD-CKX1 lines 21.4 and 4.3 showed significantly reduced CKX activity in stages 59 and 49, and 59, respectively. In stages 49 and 59, enzymatic activities declined to 30–38% and 48–63% of the enzymatic activity in control lines, for KO-CKX1 mutants and KD-CKX1 lines, respectively. The highest difference between silenced and knock-out mutants was detected in stage 71. While CKX activity of KO-CKX1 mutants was reduced to 9% of the control plant activity, in the KD-CKX1 lines it decreased only slightly to almost 80% of control lines CKX activity. The CKX activity was comparable between experiments in KD-CKX1 lines 5.8 and 4.3. Overall, these results confirmed the function of the KD-CKX1 construct, where silencing was the most efficient in KD-CKX1 line 5.8. In KO-CKX1 mutant lines, enzymatic activity stayed stable during development, confirming the successful knock-out of *HvCKX1* gene.

### Cytokinin Analysis

**FIGURE 4:**
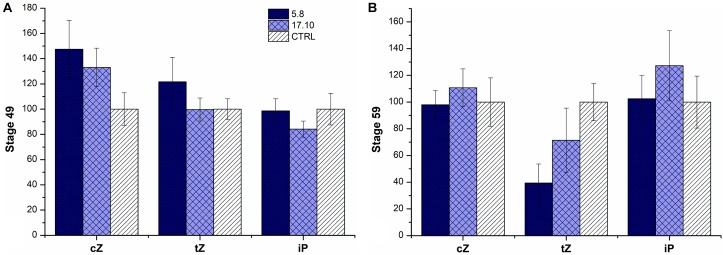
Relative cytokinin content in the spikes of two KD-CKX1 lines grown in the field. Cytokinins were measured in stage 49 [booting: first awns visible (in awned forms only)] **(A)** and stage 59 (end of heading: inflorescence fully emerged) **(B)** of two KD-CKX1 lines (5.8 and 17.10). Data are expressed as percentage of the values obtained for the control plants (CTRL) that were set up to 100%. Values are means ± SE. *cZ* includes *cZ* free base, *cZ* riboside and *cZ* monophosphate; tZ includes *tZ* free base, *tZ* riboside, *tZ* monophosphate and *tZ9*-glucoside; iP includes iP free base, iP riboside, iP monophosphate and iP9-glucoside. *c*Z, *cis*-zeatin; *t*Z, *trans*-zeatin; iP, isopentenyladenine.

**FIGURE 5:**
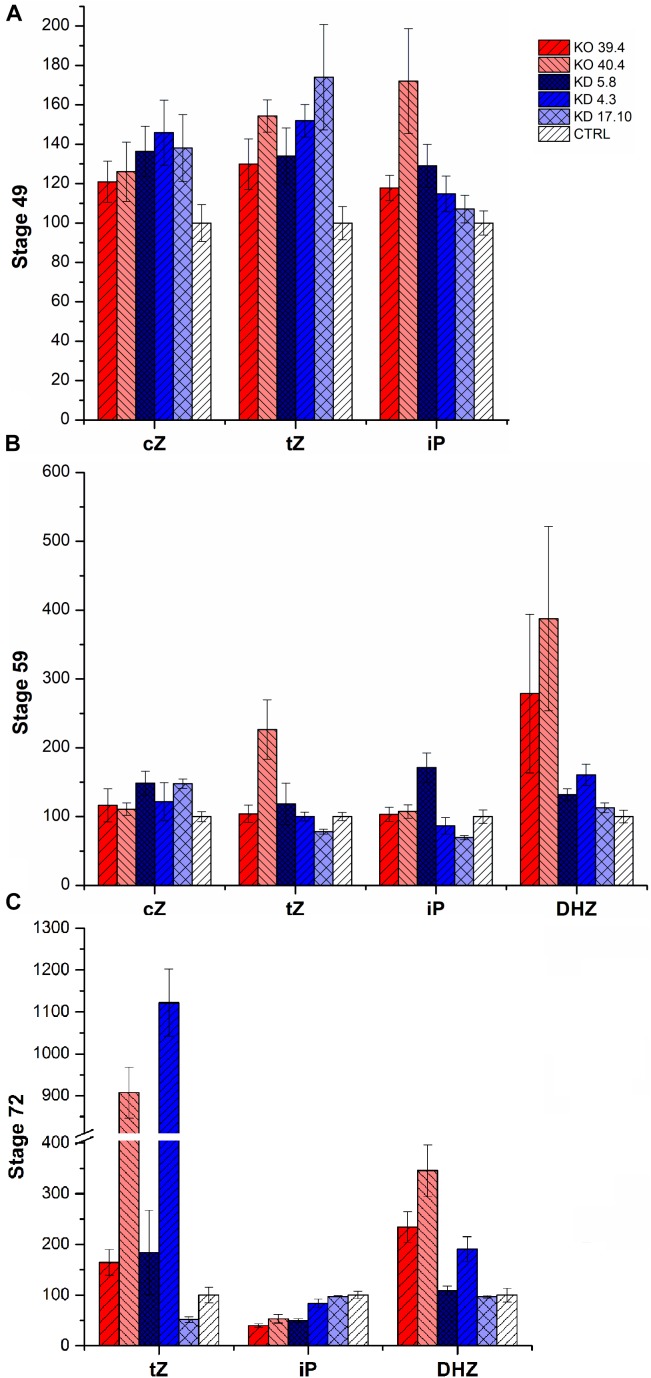
Cytokinin content in the spikes of KD-CKX1 and KO-CKX1 mutant lines during three developmental stages of plants grown in the phytotron. Cytokinins were measured in stage 49 [booting: first awns visible (in awned forms only)] **(A)**, stage 59 (end of heading: inflorescence fully emerged) **(B)** and stage 71 (watery ripe: first grains have reached half their final size) **(C)** of three KD-CKX1 lines (5.8, 17.10, and 4.3) and two KO-CKX1 mutant lines (39.4 and 40.4). Data are expressed as percentage of the values obtained for the control plants (CTRL) that were set up to 100%. Values are means ± SE. *cZ* includes *cZ* free base, *cZ* riboside and *cZ* monophosphate; *tZ* includes *tZ* free base, *tZ* riboside, *tZ* monophosphate and *tZ9*-glucoside; iP includes iP free base, iP riboside, iP monophosphate and iP9-glucoside; *c*Z, *cis*-zeatin; *t*Z, *trans*-zeatin; iP, isopentenyladenine; DHZ, dihydrozeatinriboside.

Next, we analyzed the CK content of our *HvCKX1* silenced or knock-out plants in the phytotron and under field conditions. As a control, azygous plants were used. CKs were measured in spikes of field-growing plants at stages 49 (booting) and 59 (heading); for plants grown in phytotron, spikes were harvested at stages 49, 59, and 71 (watery ripe) (Figures 4, 5).

In field experiment, there was an increase of *cis*-zeatin (*c*Z)-type CKs (including *c*Z free base, *c*Z riboside, *c*Z monophosphate) (by 50%) in both tested KD-CKX1 lines (5.8 and 17.10) and *trans*-zeatin (*t*Z)-type CKs (including tZ free base, tZ riboside, tZ monophosphate and tZ9-glucoside) in KD-CKX1 line 5.8 (by 30%) in stage 49 (Figure 4A). In stage 59, there was a small decrease in *t*Z-type CKs in both silencing lines (30%). Other types of CKs were in both KD-CKX1 lines comparable to azygous control plants (Figure 4B).

In greenhouse experiments, endogenous CK content was evaluated in 3 KD-CKX1 (5.8, 4.3, and 17.10) and two KO-CKX1 mutant lines (39.4, 40.4; Figure 5). Level of *cZ*-type CKs was increased in all tested lines (up to 50%) at stage 49, in two KD-CKX1 lines (5.8 and 17.10; up to 40%) at stage 59, and in two KD-CKX1 lines (5.8 and 17.10; double) at stage 71. Level of *t*Z-type CKs was enhanced in five lines (KO-CKX1 39.4, 40.4; KD-CKX1 5.8, 4.3, 17.10; up to 70%) at stage 49, in one line (KO-CKX1 40.4; up to 130%) at stage 59, and in five lines (KO-CKX1 39.4, 40.4, 48.1; KD-CKX1 5.8, 4.3; up to 1,000%) at stage 71. However, a decrease of *t*Z was also observed in one KD-CKX1 line (17.10) in stages 59 and 71. Isopentenyl-adenine (iP)-type of CKs (including iP free base, iP riboside, iP monophosphate and iP9-glucoside) was increased in three lines (KO-CKX1 39.4 and 40.4; KD-CKX1 5.8; by 60%) at stage 49, and in one line (KD-CKX1 5.8; by 60%) at stage 59. In stage 71, level of iP-type CKs was in all lines decreased or comparable to control plants. Dihydrozeatinriboside (DHZ)-type CKs was increased in five lines (KO-CKX1 39.4, 40.4; KD-CKX1 5.8, 4.3, 17.10; up to 250%) at stage 59, and three lines (KO-CKX1 39.4, 40.4; KD-CKX1 5.8; up to 160%) at stage 71. In stage 49, DHZ-type CKs was not detectable. The full details of the measurements can be found in Supplementary Tables S6, S7.

In general, the silencing or knock-out of *HvCKX1* gene resulted in an increase of total CK endogenous content. However, there were differences between individual CK types and lines.

### Effect of *HvCKX1* Silencing and Knock-Out on Root Growth and Shoot Biomass

**FIGURE 6:**
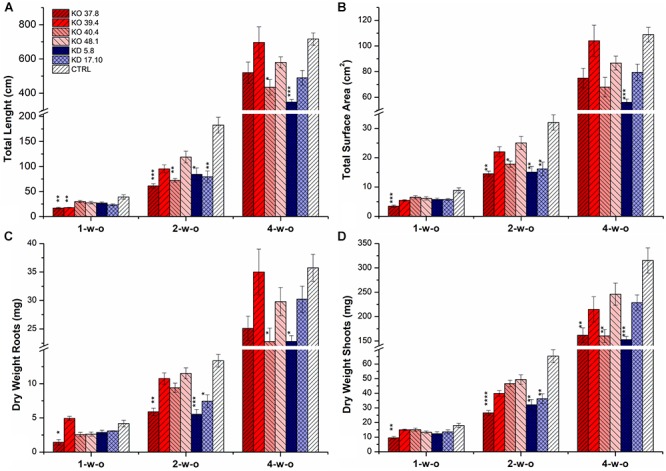
Phenotyping of the root system of different KD-CKX1 and KO-CKX1 lines growing hydroponically. Total root length **(A)**, total root surface area **(B)**, dry weight of the roots **(C)** and dry weight of the shoots **(D)** of the KD-CKX1 plants (5.8 and 17.10), KO-CKX1 mutant lines (37.8, 39.4, 40.4, and 48.1) and control plants (CTRL). Plants were grown in hydroponic conditions in Hoagland solution in phytotron (photoperiod of 24°C/16 h/light and 22°C/8 h/dark) and harvested after one (1-w-o), two (2-w-o) and four (4-w-o) weeks. Total root length and total root surface area were determined with WinRHIZO software. Values are mean ± SE (*n* = 9). A non-parametric Kruskal–Wallis Anova & Median test analysis was performed followed by a *post hoc* multiple comparison of mean rank (Statistica v. 13.3); ^∗^*p* < 0.01, ^∗∗^*p* < 0.001, ^∗∗∗^*p* < 0.0001, ^∗∗∗∗^*p* < 0.00001.

Plants of one azygous control line, two KD-CKX1 lines (5.8 and 17.10) and four KO-CKX1 mutants (37.8, 39.4, 40.4, and 48.1) were grown hydroponically to determine whether the decreased HvCKX1 activity might influence root growth. Roots and shoots of plants grown in hydroponic conditions were harvested 1, 2, and 4 weeks after germination on wet filter paper. Total root length (TRL), total surface area (TSA) and dry weight of the roots (DWR) and shoots (DWS) were determined (Figures 6A–C). Significantly reduced root growth of 1-week-old plants was seen just in KO-CKX1 mutant lines 37.8 (all parameters decreased) and 39.4 (TRL decreased). In 2-week-old plants significantly decreased root growth was observed in both KD-CKX1 lines (all parameters decreased) as well as in KO-CKX1 mutant line 37.8 (all parameters decreased) and 40.4 (TRL and TSA decreased). Analysis of 4-week-old roots showed significantly reduced roots growth in KD-CKX1 line 5.8 (all parameters decreased) and KO-CKX1 mutant lines 40.4 (TRL, DWR were decreased). Reduced shoot biomass was seen in KO-CKX1 lines 37.8 (all plants) and 48.1 (3-week-old plants) and KD-CKX1 lines 5.8 (2- and 3-week-old plants) and 17.10 (2-week-old plants) (Figure 6D). In summary, these results showed that reduced CKX activity negatively influence root growth of barley plants, but the effect varied between individual lines and it was dependent on the developmental stage of plants.

### Effect of *HvCKX1* Silencing and Knock-Out on Yield

**FIGURE 7:**
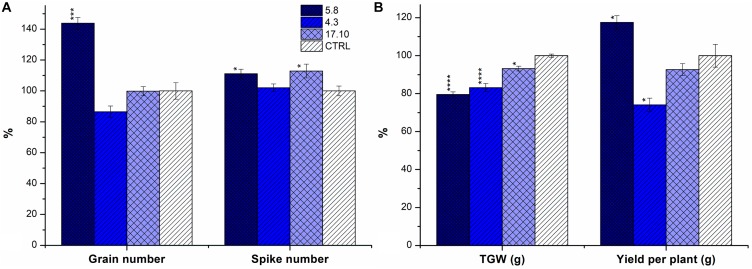
Yield parameters of KD-CKX1 lines grown in a greenhouse. Grain and spike number **(A)**; yield per plant and TGW **(B)** was calculated in KD-CKX1 lines (5.8, 4.3, and 17.10) and control plants grown in the control greenhouse conditions (photoperiod of 24°C/16 h/light and 22°C/8 h/dark) during years 2014–2015. Data are expressed as percentage (%) of the value obtained for CTRL, which was set up to 100%. Values are mean ± SE (*n* = 25). A non-parametric Kruskal–Wallis Anova & Median test analysis was performed followed by a *post hoc* multiple comparison of mean rank (Statistica v. 13.3); ^∗^*p* < 0.01, ^∗∗^*p* < 0.001, ^∗∗∗^*p* < 0.0001, ^∗∗∗∗^*p* < 0.00001.

Plants of KD-CKX1 lines 5.8, 17.10, KO-CKX1 mutant lines 37.8, 39.4, 40.4, and 48.1 and azygous control line were grown in a greenhouse (GH; 25 plants per each line) to evaluate the yield parameters such as: numbers of spikes and grains, total yield per plant and TGW. In addition, KD-CKX1 lines 5.8, 17.10 and azygous control plants were grown in the field. In field experiments spike number (50 plants per each line), TGW and yield per m^2^ were evaluated. In GH experiment, KD-CKX1 line 5.8 produced about 10% more spikes and 40% more grains per plant compared to control plants (Figure 7A) and the total yield increased to almost 120% of the control (Figure 7B). Plants of KD-CKX1 17.10 line produced about 10% more spikes, but the grain number and total yield per plants were identical to those of control plants (Figure 7). However, TGW decreased to 80 and 93% of control plants for lines KD-CKX1 5.8 and 17.10, respectively (Figure 7B).

**FIGURE 8:**
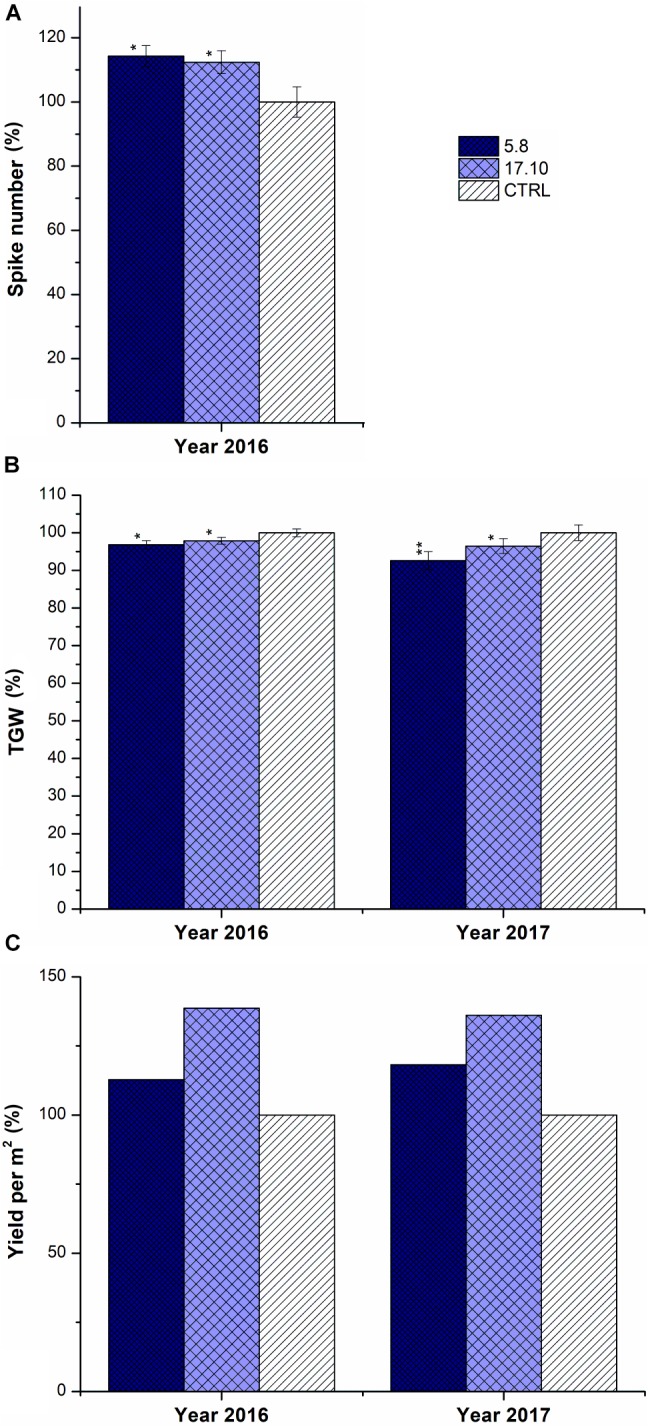
Yield parameters of KD-CKX1 lines grown under field conditions. Spike number per plant **(A)**, TGW **(B)** and yield per m^2^ **(C)** were established in KD-CKX1 lines (5.8 and 17.10) and control plants (CTRL) in 2016 and 2017. Data are expressed as percentage (%) of the value obtained for CTRL, which was set up to 100%. Values are mean ± SE (*n* = 50). A non-parametric Kruskal–Wallis Anova & Median test analysis was performed followed by a *post hoc* multiple comparison of mean rank (Statistica v. 13.3); ^∗^*p* < 0.01, ^∗∗^*p* < 0.001.

Similar to GH experiments, plants of KD-CKX1 lines 5.8 and 17.10 which were grown in the field produced up to 10% more spikes in the year 2016 (Figure 8A). Total yield per m^2^ was increased up to 120 and 139% in 2016 and up to 118 and 136% in 2017 in comparison with control plants, for lines KD-CKX1 5.8 and 17.10, respectively (Figure 8C). The TGW of KD-CKX1 plants were slightly reduced up to 97% in 2016 and to 92% in 2017 in comparison to control plants (Figure 8B).

In summary, silencing of *HvCKX1* gene negatively influences the yield of transgenic plants; although significant differences in some yield parameters were detected.

### Transcriptome Analysis of KD-CKX1 Lines

According to our hypothesis downregulation of *HvCKX1* during spike and grain development lead to larger grains, we were analyzing differentially expressed genes during booting (stage 49) and inflorescence emergence (stage 59) of two KD-CKX1 lines 5.8 and 17.10, which were the most affected in yield parameters. Eighteen libraries were prepared from the plants growing in the field (2016) and sequenced. Transcriptome sequencing produced a total of 1,119,044,252 pair-end reads, which were mapped to the reference genome of barley cv. Morex. Of the total 26,066 annotated genes, 108 and 45 genes were significantly altered (adjusted *P*-value ≤ 0.01; -2 ≤ log2 Fold Change ≥ 2) in their expression compared to control, in stage 49 of KD-CKX1 lines 5.8 and 17.10, respectively (Supplementary Table S8). In stage 59, 151, and 118 genes were significantly changed compared to control (adjusted *P*-value ≤ 0.01; -2 ≤ log2 Fold Change ≥ 2), for lines KD-CKX1 5.8 and 17.10, respectively (Supplementary Table S9). We found transcripts significantly up- or down-regulated in the two chosen lines and developmental stages. If one compares the differentially altered genes between the two stages only 10% for line 5.8 are identical. For the second line, 17.10, the expression of 29% of the transcripts present was identically altered in both stages of spike development. (adjusted *P*-value ≤ 0.01; -2 ≤ log2 Fold Change ≥ 2) for stages 49 and 59, respectively (Table 2). Among the most upregulated genes in both stages belong genes functionally annotated to play a role in RNA and DNA regulation, and hormone metabolism. The most down-regulated genes were functionally categorized into transport, abiotic stress response, peroxidases, metal-handling, signaling and others (Table 2). Four of the most differentially expressed genes, one potassium transporter, pectin methylesterase inhibitor, FMN-oxidoreductase and replication protein A (for stages 49 and 59) were verified in lines 5.8 and 17.10 by qPCR (Supplementary Tables S8, S9), confirming the results from RNAseq analysis.

**Table 2:** Functional annotation of most differentially expressed genes (adjusted *p*-value ≤ 0.01) generated according to Mercator tool (Lohse et al., 2014) in transgenic barley spikes of KD-CKX1 lines (5.8 and 17.10) compared to control plants, collected in different development stages 49 (booting) and 59 (heading), according to BBCH code for cereals.

BIN (class)	Description	Number of affected genes
**Stage 49 (booting: first awns visible (in awned forms only))**		

**Upregulated**		
17	Hormone metabolism: jasmonate synthesis/degradation	1
26	Miscellaneous: protease inhibitor/seed storage/lipid transfer protein	1
27	RNA: regulation of transcription	2
28	DNA: regulation; repair	2
35	Not assigned: unknown; transposon and retrotransposon protein	11
**Downregulated**		
20	Stress: abiotic	1
26	Miscellaneous: gluco-, galacto-, and mannosidases	1
35	Not assigned: no ontology, pentatricopeptide repeat-containing protein	1

**Stage 59 (end of heading: inflorescence fully emerged)**		

**Upregulated**		
20	Stress: biotic	3
26	Miscellaneous: plastocyanin-like	1
27	RNA: regulation of transcription	3
28	DNA: regulation; repair	2
34	Transport: sugars; p- and v-ATPase’s	3
35	Not assigned: unknown; transposon and retrotransposon protein	12
**Downregulated**		
10	Cell wall: pectinesterase	1
15	Metal handling: binding, chelation and storage	3
16	Secondary metabolism: phenylpropanoids, lignin biosynthesis	4
17	Hormone metabolism: ethylene synthesis, degradation	1
20	Stress: abiotic	6
21	Redox. regulation: thioredoxin	1
22	Polyamine metabolism: degradation	1
26	Miscellaneous: peroxidases; glutathione S transferases; oxidases – copper, flavone; O-methyl transferases	16
27	RNA: regulation of transcription	2
29	Protein degradation: cysteine protease	2
30	Signaling: receptor kinases	4
31	Cell: organization	1
33	Development: late embryogenesis abundant; storage proteins	2
34	Transport: miscellaneous; Major Intrinsic Protein; ABC transporters and multidrug resistance system; potassium; phosphate; nitrate; sugars	8
35	Not assigned: unknown; no ontology	16


*Detailed information of the genes is provided in Supplementary Tables S8, S9*.

## Discussion

In cereals yield results from the number of spikelets per unit crop area, number grains per spikelet and grain size. While number of spikelets and number of grains are determined very early in barley development, grain size can be influenced at later stages of plant development (Sreenivasulu and Schnurbusch, 2012). Endogenous cytokinin is one of such grain size influencing factor. Indeed, several studies confirmed that the amount of cytokinin is a limiting factor for yield (for review see Jameson and Song, 2016).

### Silencing or Knock-Out of *HvCKX1* Influence Endogenous *HvCKX1* Expression

In order to increase cytokinin during grain development by abolishment of the cytokinin-degrading enzyme, CKX1, we used a reverse-genetics approach to study gene function in more detail. In the present work, we used RNAi-based silencing technique as well as the guide RNA/Cas9 system to elucidate the role of *HvCKX1* gene during grain production in barley. Our experiments aimed to specifically target the single *HvCKX1* gene. A previous study used a similar silencing approach, but with a fragment targeting a 450 bp fragment of the conserved part amongst barley *CKX* genes (Zalewski et al., 2010). Since the expression of the silencing cassette leads to the formation of double-stranded RNA, which is processed to small interfering RNA (siRNA), it cannot be excluded that other barley *CKX* genes were downregulated as well. The used silencing method employs the hairpin RNA, consisting of inverted repeat of a gene fragment separated by intron, which, with regards of sequence similarity, specifically target mRNA (Wesley et al., 2001). The effectiveness of silencing is based on length of a gene fragment, with the best suitable fragment lengths between 300 and 600 bp (Helliwell and Waterhouse, 2003). Considering the high homology between barley *CKX* genes, we used a 250 bp gene fragment, consisting of the 3′UTR, as the most variable part of the *HvCKX* sequences. To avoid undesirable silencing of non-targeted genes, it was established the rule to do not have blocks of sequence similarity of over 20 bp (Helliwell and Waterhouse, 2003), as shown in the alignment. Very efficient silencing level ranging from 45 to 93% was obtained during all developmental stages analyzed using presented *SC_*KD-CKX1 construct. It is comparable or better than data already published by different research groups, which employed the same silencing vector but targeting different sequences in the barley genome (Stanley et al., 2011; Moore, 2012). Moreover, the silenced lines obtained in the frame of our study were stable through several generations.

The significant differences in *HvCKX1* expression and activity, and thus also in phenotype, were observed between individual tested silenced lines. Since the initiation of the silencing mechanism must exceed a certain threshold to become effective (Lechtenberg et al., 2003) one would expect multi-copy plants to have a higher silencing efficiency. However, an increased expression of the transgene can also lead to the silencing of the transgene cassette and thus to the inhibition of the desired effect. This is often the case when plants become homozygous in subsequent generations and thus double their copy number.

Such differences in the transgene expression of independent lines could also be caused by transgene construct fidelity or positional effect (Matzke and Matzke, 1998; Wakimoto, 1998; Kooter et al., 1999). Both copy number analysis and PCR analysis determined that 3 silencing lines contained one copy (5.8, 4.3, and 21.4) and 1 line (17.10) two copies of the full silencing cassette, which indicated that different expression pattern and different phenotype between the line 17.10 and other lines could be related to transgene copy number. Moreover, several studies showed that transgene expression level is dependent on site of integration and that some integration events could give partial spatial expression manner resulting from heritable form of gene silencing (Kooter et al., 1999; Day et al., 2000). Despite the place of integration into the genome was not determined in the frame of the present study, we can hypothesize that the different silencing level between tested homozygous lines might be due to positional effect of random integration of T-DNA into plant genome as well as to multiple transgene copies.

To completely abolish the *HvCKX1* expression the innovative guide RNA/Cas9 system was used. This very efficient technique enables specific genome modification at target site (Jinek et al., 2012). This highly universal technology was previously successfully used in monocotyledonous plants including wheat (Cermák and Curtin, 2017) and barley (Lawrenson et al., 2015; Watanabe et al., 2016; Holme et al., 2017).

At the time of mutant generation, little information was available to select efficient guide RNAs for plants. All online tools such as Deskgen or WU-CRISPR were based on human or animal systems, being thus only partially suitable for making reliable forecasts for plants. Therefore, the previously developed transient expression test system was used for pre-validation (Budhagatapalli et al., 2016). The system relies on the fact that improperly repaired double-strand breaks restore functionality of the YFP reporter gene and its fluorescence can be evaluated as a direct signal. The efficiency of ∼93% achieved there was even higher than the guide RNA for GFP used to establish the system, which achieved an activity of 31% in barley. A guide RNA selected for GFP-transgenic tobacco reaching ∼75% efficiency in the transient expression system showed also high activity in stable transgenic tobacco plants (Schedel et al., 2017). Indeed, 12 out of 15 analyzed tobacco plants exhibited a genomic modification at the target site, corresponding to an activity of 80%.

Here we successfully generated 15 *Hvckx1* mutants using this technology and confirmed that mutations were stably inherited in the next generation. Efficiency of mutation was comparable or better than previous studies (Lawrenson et al., 2015; Watanabe et al., 2016; Holme et al., 2017; Kumar et al., 2018). This data also confirmed our results obtained with the transient expression test system and therefore underpin that it is a useful tool for validation of site-directed mutagenesis constructs. All mutations were observed at 3 bp upstream of PAM sequence and led to frameshift and creation of premature stop codons or production of non-sense protein. These data are in accordance with previous studies (Lawrenson et al., 2015; Kapusi et al., 2017). The most frequent mutation detected in primary mutants was 1-bp deletion (100%), followed by 1-bp insertion (27%) suggesting that mutation were created due to NHEJ. The fact that a 20-nucleotide long sequence used as navigator for Cas9 occurs several times in a large plant genome like barley may imply the possibility of putative off-targets. To check this fact *in silico* analysis were performed. A blast search against the barley genome revealed no potential off-target sites since all sequences that had homologies to gRNA had no NGG PAM. Therefore we did not follow this aspect in more detail.

As expected, complete knock-down of *HvCKX1* could further reduce total CKX activity in the developing spike (stage 49) or the developing grain (stage 71).

### Silencing or Knock-Out of *HvCKX1* Alter CKX Activity and Cytokinin Homeostasis of the Plant

The function of CKX proteins is to irreversibly degrade CKs, therefore downregulation or knock-down of *HvCKX1* gene should alter CK homeostasis in the barley plant. We previously demonstrated that *HvCKX1* expression peaks in older leaves, roots and developing grain (Mrízová et al., 2013). Consequently, CKX activity and CK levels were measured in this developmental stages. The CKX activity was downregulated in both KD-CKX1 and KO-CKX1 mutant lines. However, differences between silenced and mutated lines were observed in the stage 71 (up to 91% comparing to 20% decrease for KO-CKX1 and KD-CKX1 lines, respectively). These results indicated that both silencing and knock-out of *HvCKX1* gene were an efficient strategy to alter endogenous CK content in barley.

CK content was measured in plant material of KD-CKX1 lines 5.8 and 17.10, collected from field (same as was later used for RNAseq analysis) as well as in plants of KD-CKX1 and KO-CKX1 mutant lines grown in phytotron in control conditions. We could observe that the total CK content increased during barley development in all analyzed lines and control. However, differences between individual cytokinin forms were noticeable. For example, the content of *cZ*-type CKs decreased during development contrary to *tZ*-type CKs, which increased. This observation is in the correlation with previous study on barley, that suggested main role of *cZ* in early stage of kernel development and *tZ* in later stages (Powell et al., 2013). Moreover, total CK content was higher in most of the KD-CKX1 and KO-CKX1 mutant lines during all tested stages in comparison to control plants. The increase of CK content was mainly related to the accumulation of *cZ*- and *tZ*-type CKs, which are predicted as the most important CK forms at the stage of kernel development (Powell et al., 2013). Similar trends were observed between field- and phytotron-grown plants, especially in stage 49; however, small differences were noticed in stage 59. These could be explained by different environmental conditions in both experiments. Indeed, CKs are known to be influenced by abiotic and biotic stresses (Bielach et al., 2017), which could led to changes in their level between plants grown in the field and in the phytotron.

### Reduced CKX Activity Influence Root System and Plant Productivity

The alteration of endogenous content by mean of silencing or knocking-out *HvCKX1* gene resulted into two important phenotypic trait changes in barley. Firstly, the root system of KD-CKX1 and KO-CKX1 mutants was inhibited, and secondly, plant productivity of KD-CKX1 lines was enhanced compared to the WT plants. It is well known that CK negatively influence the root growth (Werner et al., 2003). In our study, alteration of the transgenic root system architecture was markedly obvious at 2–4th week after germination in both KD-CKX1 and KO-CKX1 mutant lines. This corresponds well with the temporal regulation of *HvCKX1* gene expression in the barley roots, as previously shown (Mrízová et al., 2013). Thus, the negative effect of *HvCKX1* silencing and knock-out on root development is the strongest at these stages, decreasing later during root development, when plants are most probably able to compensate the leakage of CK degradation.

Studies on Arabidopsis *ckx3ckx5* double mutant (Bartrina et al., 2011) and Habataki variety of rice with non-functional *OsCKX2* gene (Ashikari et al., 2005) revealed that higher accumulation of CKs in the inflorescence meristem had a positive effect on plant yield as a consequence of increased production of reproductive organs. In our study, *HvCKX1* silencing and knocking-out increased the production of spikes, resulting in higher yield due to higher grain number. However, the weight of thousands grains was decreased. These results were observed with KD-CKX1 plants grown both in greenhouse and in field. Hence, the mechanisms how CKs regulate grain yield in transgenic barley seem to be different from that seen in rice or Arabidopsis. Increased tillering could be positively affected by higher CK amount due to its decreased degradation. Spring barley starts to produce tillers within 2–3 weeks after germination (Anderson et al., 1995), which, as already mentioned, correspond with the maximal expression of *HvCKX1* gene in the roots. This suggested a transfer of CK from roots to shoots. That hypothesis is supported by the predicted apoplastic localization of HvCKX1 as well as the fact, that CKs are mobile phytohormones, which are transported through the xylem (Kudo et al., 2010). Whereas in previous possible transport system for CKs were characterized in Arabidopsis (Gillissen et al., 2000; Bürkle et al., 2003; Cedzich et al., 2008), such information is still missing for crops, such as barley.

Although spike and grain number increased in KD-CKX1 lines, the average of thousand grain weight decrease to 80% of the control plants mass. This result is in agreement with previous study dealing with three barley cultivars (Powell et al., 2013). Indeed, the cultivar with the highest grain number had the highest CK content at the stage of starch accumulation, nevertheless, the same cultivar had the lowest average grain mass compare to other cultivars (Powell et al., 2013). However, mechanisms of regulation of grain yield by CKs remain still unexplained.

A contradictory study showed that silencing of the *HvCKX1* gene led to both increased grain number and increased thousand grain weight (Zalewski et al., 2010). A possible explanation to this discrepancy might reside in the specificity of the silencing cassette used in the two different studies, and the lack of correlation between *HvCKX1* transcript accumulation and CKX activity. Whereas we designed a silencing cassette to specifically target *HvCKX1* gene, this was not the case in the other study. Also, a higher decrease in the overall CKX activity in the study of Zalewski et al. (2010) compared to our study might indicate that other *CKX* genes were also probably targeted to silencing. Therefore the possible decreased expression level of other *CKX* genes might have influenced the CK homeostasis in the whole plant resulting in different phenotype. Study of Zalewski et al. (2010) also presented data obtained from heterozygous T0 and T1 generations and no information concerning ploidy of plants was given; also the results might have been misinterpreted.

### Differentially Regulated Transcripts Identified by RNA-seq

To get deeper insights in the regulatory network of CK-dependent grain development, RNA-seq analysis in samples from KD-CKX1 lines of stages 49 [booting: first awns visible (in awned forms only)] and 59 (end of heading: inflorescence fully emerged) were analyzed. The number of differentially expressed genes in stage 59 was almost twice as higher as in stage 49. Stage 59 is characterized by endosperm development and differentiation. Endosperm provides the nutrients to the developing and germinating embryo and thus participated in total grain mass (Sabelli and Larkins, 2009). Our analysis revealed the downregulation at stage 59 of genes encoding major nutrient transporters (phosphate; nitrate, and potassium). Decreased nutrients flow due to decreased transport probably led to decreased grain mass. Such a negative regulation of nutrients transporters by CKs has already been reported in Arabidopsis (Brenner et al., 2005; Sakakibara et al., 2006; Nam et al., 2012). Beside the genes coding for transporters also genes coding for seed storage proteins, pectinesterase inhibitor and lignin biosynthesis enzyme were downregulated at the same stage. The mentioned seed storage proteins are predicted to belong to protein superfamily of cupins, which have, similarly to pectinesterase inhibitor and lignin, a role in cell wall structure (Dunwell, 1998; Jolie et al., 2010). The composition of cell wall determines it susceptibility toward nutrients. Additionally, correlation between cell wall-related enzymes and grain biomass was previous showed in barley (Ghaffari et al., 2016; Zhang et al., 2016). Thus, these genes most probably participate in the resulting phenotype of our KD-CKX1 lines.

Among genes, which were significantly upregulated, we identified genes coding for TCP transcription factor and plantacyanin, a plastocyanin-like protein. TCP domain was previously found in proteins which have function in cell division (Cubas et al., 1999). They have been characterized to play a role during formation of shoot meristem and development of shoot lateral organs (Koyama et al., 2007). Plantacynin has role in reproduction through regulation of pollination. In Arabidopsis, plants overexpressing this gene had reduced seed set (Dong et al., 2005). However, roles of TCP transcription factor and plantacyanin in crop plants could differ from that described in Arabidopsis; therefore additional investigation on barley or other crops would be necessary to reveal their connection with CKs and regulation of grain production.

In both tested stages we also observed the downregulation of genes encoding proteins related to abiotic stress. Crosstalk between CK and abiotic stress is well known (reviewed in Bielach et al., 2017). Previous studies on Arabidopsis, tobacco and barley, where the endogenous content of CKs was modified through regulation of *IPT* or *CKX* genes showed a negative role of CK in response to stress (Nishiyama et al., 2011; Macková et al., 2013; Pospíšilová et al., 2016).

We initially hypothesized that as the *HvCKX1* gene expression reaches a maximum in aleurone layer and mature embryos, the accumulation of endogenous CKs resulting from the silencing of this gene would increase grain filling and consequently the grain weight. However, our data contradict to proposed hypothesis. Enhanced CK level due to the *HvCKX1* silencing increased tillering but decreased grain weight. Our results suggested that as the role of CKs in this process integrates several mechanisms, including regulation of cell division, nutrient transport and cell wall composition. Nevertheless, additional studies of grain composition are necessary to support our findings. Our stable transgenic lines can serves as a potential tool to study local CK maxima and their effect on productivity under different conditions, e.g., limited nitrogen supply or water deficiency. As the barley is important crop model for Triticeae tribe, such experiments which will follow CK alteration in more temporarily and spatially regulated manner are desirable.

## Conclusion

In the present study, we successfully generated homozygous transgenic plants with silenced and knock-out *HvCKX1* gene and decreased accumulation of HvCKX1 protein, which is an important negative regulator of CK content especially in developing grains and roots. As expected, decreased accumulation of HvCKX1 caused probably a local accumulation of CKs which resulted in reduced development of the root system. Unexpectedly, higher productivity of the prepared KD-CKX1 lines was achieved by formation of more tillers and subsequently more grains. Such a trait induced by a single gene knock-down/out could be beneficial for improvement of crop plants by the genetic manipulation. However, thousand grains weight decreased in *HvCKX1* silenced transgenic plants in comparison to the control plants, indicating that mechanisms by which the CKs regulate grain production are very complex employing macronutrient transporters, cell wall modification enzymes, regulators of cell division, etc. Additional studies exploiting genetic manipulation with *CKX* genes in barley are necessary to obtain highly profitable barley plants, which will produce the grains of at least the same quality as the donor Golden Promise plants.

Moreover, we used the novel techniques of genome editing employing guide RNA/Cas9 system and successfully knocked-out the target gene. This powerful tool together with obtained findings open the new possibilities to modify the grain yield in crops, which is with regards of global climate changes and intensively growing population desirable.

## Author Contributions

KH, GH, PT, and PV performed the experiments and analyzed the data. PG and VB conceived the study. KH, GH, VB, and PG wrote the manuscript. All authors read and agreed at the last version of the manuscript.

## Conflict of Interest Statement

The authors declare that the research was conducted in the absence of any commercial or financial relationships that could be construed as a potential conflict of interest.

## Footnotes

1 www.bract.org

2 https://www.deskgen.com/guidebook/ki.html

3 http://www.idnagenetics.com

4 http://webblast.ipk-gatersleben.de/barley_ibsc/viroblast.php

## References

[B1] Anderson P. M. Oelke E. A. Simmons S. R. (1995). *Growth and Development Guide for Spring Barley.* St. Paul, MN: University of Minnesota Extension Service.

[B2] Ashikari M. Sakakibara H. Lin S. Yamamoto T. Takashi T. Nishimura A. (2005). Plant science: cytokinin oxidase regulates rice grain production. *Science* 309 741–745. 10.1126/science.1113373 15976269

[B3] Bartrina I. Otto E. Strnad M. Werner T. Schmülling T. (2011). Cytokinin regulates the activity of reproductive meristems, flower organ size, ovule formation, and thus seed yield in *Arabidopsis thaliana*. *Plant Cell* 23 69–80. 10.1105/tpc.110.079079 21224426 PMC3051259

[B4] Bielach A. Hrtyan M. Tognetti V. B. (2017). Plants under stress: involvement of auxin and cytokinin. *Int. J. Mol. Sci.* 18:E1427. 10.3390/ijms18071427 28677656 PMC5535918

[B5] Bieleski R. L. (1964). The problem of halting enzyme action when extracting plant tissues. *Anal. Biochem.* 9 431–442. 10.1016/0003-2697(64)90204-0 14239480

[B6] Bradford M. M. (1976). A rapid and sensitive method for the quantitation of microgram quantities of protein utilizing the principle of protein-dye binding. *Anal. Biochem.* 72 248–254. 10.1016/0003-2697(76)90527-3 942051

[B7] Brenner W. G. Romanov G. A. Köllmer I. Bürkle L. Schmülling T. (2005). Immediate-early and delayed cytokinin response genes of *Arabidopsis thaliana* identified by genome-wide expression profiling reveal novel cytokinin-sensitive processes and suggest cytokinin action through transcriptional cascades. *Plant J.* 44 314–333. 10.1111/j.1365-313X.2005.02530.x 16212609

[B8] Budhagatapalli N. Schedel S. Gurushidze M. Pencs S. Hiekel S. Rutten T. (2016). A simple test for the cleavage activity of customized endonucleases in plants. *Plant Methods* 12:18. 10.1186/s13007-016-0118-6 26962325 PMC4784412

[B9] Bürkle L. Cedzich A. Döpke C. Stransky H. Okumoto S. Gillissen B. (2003). Transport of cytokinins mediated by purine transporters of the PUP family expressed in phloem, hydathodes, and pollen of Arabidopsis. *Plant J.* 34 13–26. 10.1046/j.1365-313X.2003.01700.x 12662305

[B10] Cedzich A. Stransky H. Schulz B. Frommer W. B. (2008). Characterization of cytokinin and adenine transport in Arabidopsis cell cultures. *Plant Physiol.* 148 1857–1867. 10.1104/pp.108.128454 18835995 PMC2593678

[B11] Cermák C. Curtin S. J. (2017). “Design and assembly of CRISPR/Cas9 reagents for gene knockout, targeted insertion, and replacement in wheat,” in *Wheat Biotechnology: Methods and Protocols*, eds Bhalla P. L. Singh M. B. (Berlin: Springer Science+Business Media LLC), 187–212. 10.1007/978-1-4939-7337-8 28913802

[B12] Cubas P. Lauter N. Doebley J. Coen E. (1999). The TCP domain: a motif found in proteins regulating plant growth and development The TCP domain: a motif found in proteins regulating plant growth and development. *Plant J.* 18 215–222. 10.1046/j.1365-313X.1999.00444.x 10363373

[B13] Day C. D. Lee E. Kobayashi J. Holappa L. D. Albert H. Ow D. W. (2000). Transgene integration into the same chromosome location can produce alleles that express at a predictable level or alleles that are differentially silenced. *Genes Dev.* 14 2869–2880. 10.1101/gad.849600 11090134 PMC317066

[B14] Dobin A. Davis C. A. Schlesinger F. Drenkow J. Zaleski C. Jha S. (2013). STAR: ultrafast universal RNA-seq aligner. *Bioinformatics* 29 15–21. 10.1093/bioinformatics/bts635 23104886 PMC3530905

[B15] Dong J. Kim S. T. Lord E. M. (2005). Plantacyanin plays a role in reproduction in Arabidopsis. *Plant Physiol.* 138 778–789. 10.1104/pp.105.063388 15908590 PMC1150396

[B16] Dunwell J. M. (1998). Cupins: a new superfamily of functionally diverse proteins that include germins and plant storage proteins. *Biotechnol. Genet. Eng. Rev.* 15 1–32. 10.1080/02648725.1998.10647950 9573603

[B17] Edwards K. Johnstone C. Thompson C. (1991). A simple and rapid method for the preparation of plant genomic DNA for PCR analysis. *Nucleic Acids Res.* 19:1349 10.1093/nar/19.6.1349 PMC333874 2030957

[B18] Enz M. Dachler C. H. (1997). *Compendium of Growth Stage Identification Keys for Mono- and Dicotyledonous Plants. Extended BBCH Scale.* Available at: http://www.gartneriraadgivningen.dk/upl/website/bbch-skala/scaleBBCH.pdf

[B19] Frébort I. Šebela M. Galuszka P. Werner T. Schmülling T. Pec P. (2002). Cytokinin oxidase/cytokinin dehydrogenase assay: optimized procedures and applications. *Anal. Biochem.* 306 1–7. 10.1006/abio.2002.5670 12069407

[B20] Gan S. Amasino R. M. (1995). Inhibition of leaf senescence by autoregulated production of cytokinin. *Science* 270 1986–1988. 8592746 10.1126/science.270.5244.1986

[B21] Ghaffari M. R. Shahinnia F. Usadel B. Junker B. Schreiber F. Sreenivasulu N. (2016). The metabolic signature of biomass formation in barley. *Plant Cell Physiol.* 57 1943–1960. 10.1093/pcp/pcw117 27388338

[B22] Gillissen B. Burkle L. Andre B. Kuhn C. Rentsch D. Brandl B. (2000). A new family of high-affinity transporters for adenine, cytosine, and purine derivatives in Arabidopsis. *Plant Cell* 12 291–300. 10.1105/tpc.12.2.291 10662864 PMC139765

[B23] Gregersen P. L. Culetic A. Boschian L. Krupinska K. (2013). Plant senescence and crop productivity. *Plant Mol. Biol.* 82 603–622. 10.1007/s11103-013-0013-8 23354836

[B24] Harwood W. Bartlett J. G. Alves S. C. Perry M. Smedley M. A. Leyl L. (2009). “Barley transformation using agrobacterium-mediated techniques,” in *Transgenic Wheat, Barley and Oats. Methods in Molecular Biology^TM^ (Methods and Protocols)* Vol. 478 eds Jones H. Shewry P. (New York, NY: Humana Press), 10.1007/978-1-59745-379-0_9 19009444

[B25] Helliwell C. Waterhouse P. (2003). Constructs and methods for high-throughput gene silencing in plants. *Methods* 30 289–295. 10.1016/S1046-2023(03)00036-7 12828942

[B26] Hensel G. Kastner C. Oleszczuk S. Riechen J. Kumlehn J. (2009). Agrobacterium-mediated gene transfer to cereal crop plants: current protocols for barley, wheat, triticale, and maize. *Int. J. Plant Genomics* 2009:835608. 10.1155/2009/835608 19584907 PMC2699555

[B27] Hensel G. Valkov V. Middlefell-Williams J. Kumlehn J. (2008). Efficient generation of transgenic barley: the way forward to modulate plant-microbe interactions. *J. Plant Physiol.* 165 71–82. 10.1016/j.jplph.2007.06.015 17905476

[B28] Hoagland D. R. Arnon D. I. (1950). The water-culture method for growing plants without soil. *Calif. Agric. Exp. Stn. Circ.* 347:32.

[B29] Holme I. B. Wendt T. Gil-Humanes J. Deleuran L. C. Starker C. G. Voytas D. F. (2017). Evaluation of the mature grain phytase candidate HvPAPhy_a gene in barley (*Hordeum vulgare* L.) using CRISPR/Cas9 and TALENs. *Plant Mol. Biol.* 95 111–121. 10.1007/s11103-017-0640-6 28755320

[B30] Hruz T. Laule O. Szabo G. Wessendorp F. Bleuler S. Oertle L. (2008). Genevestigator V3: a reference expression database for the meta-analysis of transcriptomes. *Adv. Bioinformatics* 2008:420747. 10.1155/2008/420747 19956698 PMC2777001

[B31] Jameson P. E. Song J. (2016). Cytokinin: a key driver of seed yield. *J. Exp. Bot.* 67 593–606. 10.1093/jxb/erv461 26525061

[B32] Jinek M. Chylinski K. Fonfara I. Hauer M. Doudna J. A. Charpentier E. (2012). A programmable dual-RNA – guided DNA endonuclease in adaptive bacterial immunity. *Science* 337 816–822. 10.1126/science.1225829 22745249 PMC6286148

[B33] Jolie R. P. Duvetter T. Van Loey A. M. Hendrickx M. E. (2010). Pectin methylesterase and its proteinaceous inhibitor: a review. *Carbohydr. Res.* 345 2583–2595. 10.1016/j.carres.2010.10.002 21047623

[B34] Jordi W. Schapendonk A. Davelaar E. Stoopen G. M. Pot C. S. De Visser R. (2000). Increased cytokinin levels in transgenic P(SAG12)-IPT tobacco plants have large direct and indirect effects on leaf senescence, photosynthesis and N partitioning. *Plant Cell Environ.* 23 279–289. 10.1046/j.1365-3040.2000.00544.x

[B35] Kapusi E. Corcuera-Gómez M. Melnik S. Stoger E. (2017). Heritable genomic fragment deletions and small indels in the putative ENGase gene induced by CRISPR/Cas9 in barley. *Front. Plant Sci.* 8:540. 10.3389/fpls.2017.00540 28487703 PMC5404177

[B36] Kieber J. J. Schaller G. E. (2018). Cytokinin signaling in plant development. *Development* 145:dev149344. 10.1242/dev.149344 29487105

[B37] Kooter J. M. Matzke M. A. Meyer P. (1999). Listening to the silent genes: transgene silencing, gene regulation and pathogen control. *Trends Plant Sci.* 4 340–347. 10.1016/S1360-1385(99)01467-3 10462766

[B38] Koyama T. Furutani M. Tasaka M. Ohme-Takagi M. (2007). TCP transcription factors control the morphology of shoot lateral organs via negative regulation of the expression of boundary-specific genes in Arabidopsis. *Plant Cell* 19 473–484. 10.1105/tpc.106.044792 17307931 PMC1867346

[B39] Kudo T. Kiba T. Sakakibara H. (2010). Metabolism and long-distance translocation of cytokinins. *J. Integr. Plant Biol.* 52 53–60. 10.1111/j.1744-7909.2010.00898.x 20074140

[B40] Kumar N. Galli M. Ordon J. Stuttmann J. Kogel K.-H. Imani J. (2018). Further analysis of barley MORC1 using a highly efficient RNA-guided Cas9 gene editing system. *Plant Biotechnol. J.* 16 1892–1903. 10.1111/pbi.12924 29577542 PMC6181210

[B41] Lawrenson T. Shorinola O. Stacey N. Li C. Østergaard L. Patron N. (2015). Induction of targeted, heritable mutations in barley and *Brassica oleracea* using RNA-guided Cas9 nuclease. *Genome Biol.* 16:258. 10.1186/s13059-015-0826-7 26616834 PMC4663725

[B42] Lechtenberg B. Schubert D. Forsbach A. Gils M. Schmidt R. (2003). Neither inverted repeat T-DNA configurations nor arrangements of tandemly repeated transgenes are sufficient to trigger transgene silencing. *Plant J.* 34 507–517. 10.1046/j.1365-313X.2003.01746.x 12753589

[B43] Liao Y. Smyth G. K. Shi W. (2014). FeatureCounts: an efficient general purpose program for assigning sequence reads to genomic features. *Bioinformatics* 30 923–930. 10.1093/bioinformatics/btt656 24227677

[B44] Lin Y. J. Cao M. L. Xu C. G. Chen H. Wei J. Zhang Q. F. (2002). Cultivating rice with delaying led-senescence by P-SAG12-IPT gene transformation. *Acta Bot. Sin.* 44 1333–1338.

[B45] Liu L. Zhou Y. Szczerba M. W. Li X. Lin Y. (2010). Identification and application of a rice senescence-associated promoter. *Plant Physiol.* 153 1239–1249. 10.1104/pp.110.157123 20439547 PMC2899913

[B46] Lohse M. Nagel A. Herter T. May P. Schroda M. Zrenner R. (2014). Mercator: a fast and simple web server for genome scale functional annotation of plant sequence data. *Plant Cell Environ.* 37 1250–1258. 10.1111/pce.12231 24237261

[B47] Love M. I. Huber W. Anders S. (2014). Moderated estimation of fold change and dispersion for RNA-seq data with DESeq2. *Genome Biol.* 15:550. 10.1186/s13059-014-0550-8 25516281 PMC4302049

[B48] Macková H. Hronková M. Dobrá J. Turečková V. Novák O. Lubovská Z. (2013). Enhanced drought and heat stress tolerance of tobacco plants with ectopically enhanced cytokinin oxidase/dehydrogenase gene expression. *J. Exp. Bot.* 64 2805–2815. 10.1093/jxb/ert131 23669573 PMC3741687

[B49] Mascher M. Gundlach H. Himmelbach A. Beier S. Twardziok S. O. Wicker T. (2017). A chromosome conformation capture ordered sequence of the barley genome. *Nature* 544 427–433. 10.1038/nature22043 28447635

[B50] Matzke A. J. M. Matzke M. A. (1998). Position effects and epigenetic silencing of plant transgenes. *Curr. Opin. Plant Biol.* 1 142–148. 10.1016/S1369-5266(98)80016-2 10066569

[B51] Moore T. E. (2012). *Are Barley Dwarfing Genes Important in Tolerance to Abiotic Stress?* Ph.D. Thesis, University of East Anglia, Norwich.

[B52] Mrízová K. Jiskrová E. Vyroubalovš Š Novák O. Ohnoutková L. Pospišilová H. (2013). Overexpression of cytokinin dehydrogenase genes in barley (*Hordeum vulgare* cv. golden promise) fundamentally affects morphology and fertility. *PLoS One* 8:e79029. 10.1371/journal.pone.0079029 24260147 PMC3829838

[B53] Nam Y. J. Tran L. S. P. Kojima M. Sakakibara H. Nishiyama R. Shin R. (2012). Regulatory roles of cytokinins and cytokinin signaling in response to potassium deficiency in Arabidopsis. *PLoS One* 7:e47797. 10.1371/journal.pone.0047797 23112848 PMC3480408

[B54] Nishiyama R. Watanabe Y. Fujita Y. Le D. T. Kojima M. Werner T. (2011). Analysis of cytokinin mutants and regulation of cytokinin metabolic genes reveals important regulatory roles of cytokinins in drought, salt and abscisic acid responses, and abscisic acid biosynthesis. *Plant Cell* 23 2169–2183. 10.1105/tpc.111.087395 21719693 PMC3160038

[B55] Novák O. Hauserová E. Amakorová P. Doležal K. Strnad M. (2008). Cytokinin profiling in plant tissues using ultra-performance liquid chromatography-electrospray tandem mass spectrometry. *Phytochemistry* 69 2214–2224. 10.1016/j.phytochem.2008.04.022 18561963

[B56] Pallotta M. A. Graham R. D. Langridge P. Sparrow D. H. B. Barker S. J. (2000). RFLP mapping of manganese efficiency in barley. *Theor. Appl. Genet.* 101 1100–1108. 10.1007/s001220051585

[B57] Pospíšilová H. Jiskrová E. Vojta P. Mrízová K. Kokáš F. Čudejková M. M. (2016). Transgenic barley overexpressing a cytokinin dehydrogenase gene shows greater tolerance to drought stress. *N. Biotechnol.* 33 692–705. 10.1016/j.nbt.2015.12.005 26773738

[B58] Powell A. F. Paleczny A. R. Olechowski H. Emery R. J. (2013). Changes in cytokinin form and concentration in developing kernels correspond with variation in yield among field-grown barley cultivars. *Plant Physiol. Biochem.* 64 33–40. 10.1016/j.plaphy.2012.12.010 23352907

[B59] Puchta H. Fauser F. (2014). Synthetic nucleases for genome engineering in plants: prospects for a bright future. *Plant J.* 78 727–741. 10.1111/tpj.12338 24112784

[B60] R Development Core Team (2008). *R: A Language and Environment for Statistical Computing.* Vienna: R Foundation for Statistical Computing Available at: http://www.r-project.org

[B61] Sabelli P. A. Larkins B. A. (2009). The development of endosperm in grasses. *Plant Physiol.* 149 14–26. 10.1104/pp.108.129437 19126691 PMC2613697

[B62] Sakakibara H. (2006). CYTOKININS: activity, biosynthesis, and translocation. *Annu. Rev. Plant Biol.* 57 431–449. 10.1146/annurev.arplant.57.032905.105231 16669769

[B63] Sakakibara H. Takei K. Hirose N. (2006). Interactions between nitrogen and cytokinin in the regulation of metabolism and development. *Trends Plant Sci.* 11 440–448. 10.1016/j.tplants.2006.07.004 16899391

[B64] Schedel S. Pencs S. Hensel G. Müller A. Rutten T. Kumlehn J. (2017). RNA-guided Cas9-induced mutagenesis in tobacco followed by efficient genetic fixation in doubled haploid plants. *Front. Plant Sci.* 7:1995. 10.3389/fpls.2016.01995 28101094 PMC5209389

[B65] Shapiro H. M. (2003). *Practical Flow Cytometry*, 4th Edn. Hoboken, NJ: Wiley-liss.

[B66] Sreenivasulu N. Schnurbusch T. (2012). A genetic playground for enhancing grain number in cereals. *Trends Plant Sci.* 17 91–101. 10.1016/j.tplants.2011.11.003 22197176

[B67] Stanley D. Rejzek M. Naested H. Smedley M. Otero S. Fahy B. (2011). The role of -glucosidase in germinating barley grains. *Plant Physiol.* 155 932–943. 10.1104/pp.110.168328 21098673 PMC3032477

[B68] Sýkorová B. Kurešová G. Daskalova S. Trčková M. Hoyerová K. Raimanová I. (2008). Senescence-induced ectopic expression of the A. tumefaciens ipt gene in wheat delays leaf senescence, increases cytokinin content, nitrate influx, and nitrate reductase activity, but does not affect grain yield. *J. Exp. Bot.* 59 377–387. 10.1093/jxb/erm319 18267946

[B69] Voytas D. F. (2013). Plant genome engineering with sequence-specific nucleases. *Annu. Rev. Plant Biol.* 64 327–350. 10.1146/annurev-arplant-042811-105552 23451779

[B70] Wakimoto B. T. (1998). Beyond the nucleosome: epigenetic aspects of position-effect variegation in *Drosophila*. *Cell* 93 321–324. 10.1016/S0092-8674(00)81159-9 9590165

[B71] Watanabe K. Breier U. Hensel G. Kumlehn J. Schubert I. Reiss B. (2016). Stable gene replacement in barley by targeted double-strand break induction. *J. Exp. Bot.* 67 1433–1445. 10.1093/jxb/erv537 26712824 PMC4762383

[B72] Werner T. Motyka V. Laucou V. Smets R. Van Onckelen H. Schmuelling T. (2003). Cytokinin-deficient transgenic Arabidopsis plants show functions of cytokinins in the regulation of shoot and root meristem activity. *Plant Cell* 15 2532–2550. 14555694 10.1105/tpc.014928 PMC280559

[B73] Wesley S. V. Helliwell C. A. Smith N. A. Wang M. B. Rouse D. T. Liu Q. (2001). Construct design for efficient, effective and high-throughput gene silencing in plants. *Plant J.* 27 581–590. 10.1046/j.1365-313X.2001.01105.x 11576441

[B74] Zalabák D. Pospíšilová H. Šmehilová M. Mrízová K. Frébort I. Galuszka P. (2013). Genetic engineering of cytokinin metabolism: prospective way to improve agricultural traits of crop plants. *Biotechnol. Adv.* 31 97–117. 10.1016/j.biotechadv.2011.12.003 22198203

[B75] Zalewski W. Galuszka P. Gasparis S. Orczyk W. Nadolska-Orczyk A. (2010). Silencing of the HvCKX1 gene decreases the cytokinin oxidase/dehydrogenase level in barley and leads to higher plant productivity. *J. Exp. Bot.* 61 1839–1851. 10.1093/jxb/erq052 20335409

[B76] Zhang R. Tucker M. R. Burton R. A. Shirley N. J. Little A. Morris J. (2016). The dynamics of transcript abundance during cellularisation of developing barley endosperm. *Plant Physiol.* 170 1549–1565. 10.1104/pp.15.01690 26754666 PMC4775131

