# Corrigendum: Modification of Barley Plant Productivity Through Regulation of Cytokinin Content by Reverse-Genetics Approaches

**DOI:** 10.3389/fpls.2018.01996

**Published:** 2019-02-04

**Authors:** Katarína Holubová, Goetz Hensel, Petr Vojta, Petr Tarkowski, Véronique Bergougnoux, Petr Galuszka

**Affiliations:** ^1^Department of Molecular Biology, Centre of the Region Haná for Biotechnological and Agricultural Research, Faculty of Science, Palacký University, Olomouc, Czechia; ^2^Plant Reproductive Biology, Leibniz Institute of Plant Genetics and Crop Plant Research (IPK) Gatersleben, Gatersleben, Germany; ^3^Institute of Molecular and Translation Medicine, Faculty of Medicine and Dentistry, Palacký University, Olomouc, Czechia; ^4^Central Laboratories and Research Support, Centre of the Region Haná for Biotechnological and Agricultural Research, Faculty of Science, Palacký University, Olomouc, Czechia; ^5^Department of Genetic Resources for Vegetables, Medicinal and Special Plants, Crop Research Institute, Centre of the Region Haná for Biotechnological and Agricultural Research, Olomouc, Czechia

**Keywords:** cytokinin, barley, CRISPR-Cas9, silencing, yield

In the published article, there was an error in both affiliations 4 and 5. Instead of the “Laboratory of Growth Regulators, Centre of the Region Haná for Biotechnological and Agricultural Research, Palacký University, Olomouc, Czechia” and the “Institute of Experimental Botany, Academy of Science of the Czech Republic, Olomouc, Czechia”, it should be the “Central Laboratories and Research Support, Centre of the Region Haná for Biotechnological and Agricultural Research, Faculty of Science, Palacký University, Olomouc, Czechia” and the “Department of Genetic Resources for Vegetables, Medicinal and Special Plants, Crop Research Institute, Centre of the Region Haná for Biotechnological and Agricultural Research, Olomouc, Czechia.”

The authors apologize for this error and state that this does not change the scientific conclusions of the article in any way. The original article has been updated.

## Conflict of Interest Statement

The authors declare that the research was conducted in the absence of any commercial or financial relationships that could be construed as a potential conflict of interest.

