# Supplementary material for: Modification of Barley Plant Productivity Through Regulation of Cytokinin Content by Reverse-Genetics Approaches
Source: Front Plant Sci. 2018 Nov 27;9:1676. doi: 10.3389/fpls.2018.01676 (PMC6277847; doi:10.3389/fpls.2018.01676)
Supplement: Supplementary file 1 [file Table_1.pdf]

Supplementary material

Table S1. Primers used for confirmation of the presence and orientation of *SC_HvCKX1* in pBract207.

Primer	Insert orientation[(a)]	Primer orientation[(b)]	Sequence 5´to 3´
UbiProm_FW	sense	F	tgctcaccctgttgtttggtgttac
ColEI_REV	antisense	R	aaggccgcgttgctggcgtttttccat
SC1_REV	sense	R	ctcaaaggagcagtttgaatatgtcttgg
IV2_FW	antisense	F	gtaagttctgcttctacctttgat
SC1_FW	antisense	R	gtggagatgaaggacaagtacga

[(a)] Orientation of *SC_HvCKX1* in pBract207. [(b)] "F" denotes forward and "R" reverse primer orientation.
