# Supplementary material for: Modification of Barley Plant Productivity Through Regulation of Cytokinin Content by Reverse-Genetics Approaches
Source: Front Plant Sci. 2018 Nov 27;9:1676. doi: 10.3389/fpls.2018.01676 (PMC6277847; doi:10.3389/fpls.2018.01676)
Supplement: Supplementary file 2 [file Table_2.pdf]

Table S2. Primers used for generation of *HvCKX1* knockout constructs and screening of *Hvckx1* mutant lines.

Primer	Primer orientation[a]	Sequence 5´to 3´	Purpose
CKX1_RGEN1_for	F	ggcgtgcgccgtaggagacgccg	Cloning of guide RNA
CKX1_RGEN1_rev	R	aaaccggcgtctcctacggcgcac	
CKX1_Target_for	F	ggatccgtcgttcacgtgttactggtcgctctgatcaccgcggcgtctcctagaattc	Cloning of target sequence
CKX1_Target_rev	R	gaattctaggagacgccgcggtgatcagagcgaccagtaacacgtgaacgacggatcc	
GH-CKX1-target	F	accgtagcagcatctcacag	Amplification of target region
GH-CKX1-target	R	gctccaggacgttggaatc	
GH-OsU3P	F	tcagcgggtcaccagtgttg	Amplification for presence of sgRNA
GH-OsU3T	R	cagggaccatagcacaagac	
GH-UBI	F	tggttagggcccggtagttc	Amplification of ZmUBI1::Cas9 junction
GH-zCas9	R	ttaatcatgtgggccagagc	

[a] "F" denotes forward and "R" reverse primer orientation.
