# Supplementary material for: Modification of Barley Plant Productivity Through Regulation of Cytokinin Content by Reverse-Genetics Approaches
Source: Front Plant Sci. 2018 Nov 27;9:1676. doi: 10.3389/fpls.2018.01676 (PMC6277847; doi:10.3389/fpls.2018.01676)
Supplement: Supplementary file 3 [file Table_3.pdf]

**Table S3. Primers used for qPCR experiments.**

| Primer/Probe | Gene | Primer orientation [a] | Sequence 5´to 3´ |
|---|---|---|---|
| HvCKX1_FW | *HvCKX1* | F | tgtggacagtaacacagcagtttaac |
| HvCKX1_REV | *HvCKX1* | R | caaaaaataatatgctctctcgagctt |
| HvCKX1_Probe | *HvCKX1* | | tggcacggtaataagatgctctgtcgc |
| EF2_FW | Elongation factor [RG] | F | aagtcctgccgcactgtcat |
| EF2_REV | Elongation factor [RG] | R | gggcgagcttccatgtaaag |
| EF2_Probe | Elongation factor [RG] | | agcaagtcccccaacaagcataaccg |
| HORVU3Hr1G022710_FW | ATP binding [RG] | F | ctaaagggtgtggaagctgtt |
| HORVU3Hr1G022710_REV | ATP binding [RG] | R | ccctttgaggcaacatcagt |
| HORVU3Hr1G022710_Probe | ATP binding [RG] | | ccacggaggcaaagatcaggagg |
| HORVU1Hr1G061690.2_FW | Nucleic acid binding [RG] | F | cctatgtgcctcaagtctttc |
| HORVU1Hr1G061690.2_REV | Nucleic acid binding [RG] | R | cgttcttctttctccgtgttac |
| HORVU1Hr1G061690.2_Probe | Nucleic acid binding[RG] | | cctgaagctcatgcaaatctcctcc |
| MLOC_60700_FW | Replic. protein | F | caccgttctgctacctcttatg |
| MLOC_60700_REV | Replic. protein | R | ctgtgccatctgttccttctaa |
| MLOC_44678_FW | FMN oxidoreductase | F | ccgtatagagaggcattcaaagg |
| MLOC_44678_REV | FMN oxidoreductase | R | ggccactaggtcagtgtaa |
| MLOC_15580_FW | Potassium transporter | F | gcactacgtccaagtcaagag |
| MLOC_15580_REV | Potassium transporter | R | gatcctcctcacatcgttcttc |
| MLOC_65390_FW | Pectin methylesterase inhibitor | F | gctcaacatcgacttcctctc |
| MLOC_65390_REV | Pectin methylesterase inhibitor | R | tgctggttggtcaggattg |

[a] "F" denotes forward and "R" reverse primer orientation. [RG] - reference gene
