# Supplementary material for: Modification of Barley Plant Productivity Through Regulation of Cytokinin Content by Reverse-Genetics Approaches
Source: Front Plant Sci. 2018 Nov 27;9:1676. doi: 10.3389/fpls.2018.01676 (PMC6277847; doi:10.3389/fpls.2018.01676)
Supplement: Supplementary file 4 [file Table_4.pdf]

Table S4. PCR conditions used for screening of KD- and KO-CKX1 lines performed with GoTaq polymerase.

Set of primers	Initial denaturation	Cycle – 30x			Final elongation	Reaction mixture*
		Denaturation	Annealing	Elongation		
SC1_FW x IV2_FW	98°C 60 sec	98°C 10s	59°C 10 sec	72°C 40 sec	72°C 10 min	1 x reaction buffer<br>2.5 mM $MgCl_2$<br>0.1 mM dNTPs<br>0.1 µM of each primer; 2% DMSO<br>1.25 U GoTaq polymerase; 200 ng of gDNA
SC1_REV x UbiProm_FW			65°C 10 sec	72°C 30 sec		
GH-CKX1-target_FW x GH-CKX1-target_REV			58°C 10 sec	72°C 60 sec		
GH-OsU3P_FW x GH-OsU3T_REV			55°C 10 sec	72°C 60 sec		
GH-UBI_FW x GH-zCas9_REV			55°C 10 sec	72°C 90 sec		

* Final concentration in reaction used for all sets of primers
