# Supplementary material for: Modification of Barley Plant Productivity Through Regulation of Cytokinin Content by Reverse-Genetics Approaches
Source: Front Plant Sci. 2018 Nov 27;9:1676. doi: 10.3389/fpls.2018.01676 (PMC6277847; doi:10.3389/fpls.2018.01676)
Supplement: Supplementary file 5 [file Table_5.pdf]

Table S5. Relative gene expression of *HvCKX* and *HvIPT* genes in KD- and KO-CKX1 lines at BBCH stages 49 (booting: first awns visible (in awned forms only)), 59 (end of heading: inflorescence fully emerged) and 71 (watery ripe: first grains have reached half their final size).

	HvCKX2.2		*HvCKX3*		*HvCKX4*		*HvCKX5*		*HvCKX9*		*HvCKX11*		*HvIPT1*		*HvIPT5*	
	**RQ**	**SD**	**RQ**	**SD**	**RQ**	**SD**	**RQ**	**SD**	**RQ**	**SD**	**RQ**	**SD**	**RQ**	**SD**	**RQ**	**SD**
CTRL stage 49	1.00	0.00	1.00	0.00	1.00	0.00	1.00	0.00	1.00	0.00	1.00	0.00	1.00	0.00	1.00	0.00
CTRL stage 59	0.93	0.13	0.96	0.15	1.08	0.28	1.03	0.17	0.89	0.11	0.96	0.12	0.98	0.08	0.99	0.16
CTRL stage 71	0.92	0.14	0.99	0.09	1.09	0.21	1.00	0.13	1.01	0.17	1.04	0.16	1.06	0.10	1.02	0.02
**KO-CKX1 lines**																
48.1 stage 49	1.03	0.08	1.02	0.14	1.08	0.10	1.08	0.10	1.18	0.16	1.04	0.04	1.03	0.04	1.05	0.04
48.1 stage 59	0.88	0.07	1.04	0.12	1.15	0.10	0.92	0.07	0.92	0.08	0.95	0.05	0.98	0.03	1.00	0.02
48.1 stage 71	0.91	0.04	0.87	0.12	1.17	0.19	0.93	0.11	0.98	0.05	0.98	0.15	0.98	0.10	1.00	0.04
39.4 stage 49	0.96	0.11	1.11	0.11	1.09	0.10	0.99	0.09	0.99	0.10	0.96	0.04	0.97	0.04	1.00	0.04
39.4 stage 59	0.85	0.09	1.02	0.18	1.23	0.14	0.90	0.05	0.85	0.04	0.91	0.06	0.95	0.03	1.02	0.05
39.4 stage 71	0.83	0.05	0.91	0.10	1.21	0.22	0.84	0.09	0.85	0.09	0.93	0.06	0.99	0.04	0.98	0.02
40.4 stage 49	1.00	0.08	1.03	0.14	1.08	0.18	1.01	0.10	1.17	0.11	0.99	0.08	0.95	0.05	1.01	0.01
40.4 stage 59	0.85	0.13	0.94	0.06	1.12	0.18	0.85	0.09	0.86	0.10	0.91	0.19	0.93	0.11	0.95	0.06
40.4 stage 71	0.94	0.11	1.10	0.11	1.21	0.17	0.92	0.07	1.03	0.05	0.92	0.06	0.95	0.11	0.98	0.02
**KD-CKX1 lines**																
5.8 stage 49	0.95	0.09	0.84	0.08	1.03	0.18	0.83	0.04	1.01	0.11	0.91	0.11	0.90	0.08	0.92	0.01
5.8 stage 59	0.95	0.08	1.02	0.08	1.07	0.18	0.91	0.07	0.90	0.09	1.03	0.18	0.98	0.03	0.99	0.03
5.8 stage 71	0.90	0.07	0.99	0.08	1.23	0.17	0.91	0.06	0.94	0.06	0.99	0.02	1.01	0.01	1.02	0.00
17.10 stage 49	0.96	0.08	0.88	0.10	1.15	0.10	0.85	0.01	1.06	0.13	0.99	0.04	0.97	0.03	0.91	0.03
17.10 stage 59	0.87	0.09	1.02	0.11	1.29	0.17	0.87	0.03	0.82	0.05	0.93	0.01	1.01	0.02	1.06	0.03
17.10 stage 71	0.83	0.11	1.03	0.17	1.27	0.16	0.84	0.05	0.96	0.04	0.97	0.02	1.01	0.00	1.01	0.03
4.3 stage 49	0.96	0.08	0.80	0.04	1.05	0.17	0.86	0.01	0.93	0.02	0.97	0.06	0.94	0.03	0.96	0.04
4.3 stage 59	0.92	0.09	1.04	0.17	1.26	0.15	0.85	0.04	0.74	0.04	0.91	0.02	1.00	0.01	1.03	0.02
4.3 stage 71	1.02	0.11	1.03	0.11	1.21	0.10	0.83	0.03	0.96	0.05	0.97	0.06	0.95	0.11	0.98	0.02
21.4 stage 49	0.95	0.11	0.86	0.14	1.13	0.11	0.84	0.11	0.98	0.09	1.19	0.24	1.11	0.20	0.94	0.03
21.4 stage 59	0.98	0.17	0.99	0.16	1.28	0.21	0.86	0.07	0.80	0.07	1.01	0.12	1.08	0.14	1.04	0.02
21.4 stage 71	0.90	0.05	1.02	0.10	1.25	0.23	0.83	0.03	0.87	0.09	0.86	0.13	0.95	0.11	1.00	0.00

RQ - relative gene expression; SD – standard deviation; values are mean ± SD (n = 5 to 8); Normalization was done in relation to three reference genes: *Elongation factor 2* - HORVU5Hr1G116580.8; *ATP-binding gene* - HORVU3Hr1G022710; *Nucleic acid binding gene* - HORVU1Hr1G061690.2. Results were expressed as fold change relative to the expression of *HvCKX* and *HvIPT* genes in the CTRL grown in the same conditions.
