# Supplementary material for: Modification of Barley Plant Productivity Through Regulation of Cytokinin Content by Reverse-Genetics Approaches
Source: Front Plant Sci. 2018 Nov 27;9:1676. doi: 10.3389/fpls.2018.01676 (PMC6277847; doi:10.3389/fpls.2018.01676)
Supplement: Supplementary file 6 [file Table_6.pdf]

Table S6. Cytokinin content in KD-CKX1 lines 5.8 and 17.10 of stages 49 (booting: first awns visible (in awned forms only)) and 59 (end of heading: inflorescence fully emerged).

49 (booting: first awns visible (in awned forms only))																								
	**cZ**		**cZR**		**cZMP**		**tZ**		**tZR**		**tZMP**		**iP**		**iPR**		**iP9G**		**iPMP**		**DHZ**		**DHZR**	
**Line**	**Mean**	**SD**	**Mean**	**SD**	**Mean**	**SD**	**Mean**	**SD**	**Mean**	**SD**	**Mean**	**SD**	**Mean**	**SD**	**Mean**	**SD**	**Mean**	**SD**	**Mean**	**SD**	**Mean**	**SD**	**Mean**	**SD**
**5.8**	66.16	10.95	702.76	304.89	83.12	34.35	16.06	5.86	104.46	50.08	126.68	62.96	28.76	8.02	59.63	16.42	5.40	1.93	9.23	2.51	0.46	0.20	6.95	2.43
**17.10**	76.61	11.84	617.33	211.61	70.31	13.73	15.28	2.38	78.77	19.41	108.41	31.76	25.94	8.99	47.15	10.06	4.87	1.44	7.88	2.96	0.72	0.15	6.57	2.27
**CTRL**	82.87	17.63	418.66	170.13	79.92	22.28	15.88	3.38	67.98	15.48	119.27	36.44	38.13	16.25	47.47	16.44	4.90	1.97	11.51	3.91	0.48	0.18	4.71	1.37
**59 (end of heading: inflorescence fully emerged)**																								
**5.8**	158.97	39.65	327.24	100.34	30.33	8.95	504.19	264.80	313.54	248.77	74.06	45.32	8.82	4.09	27.03	12.23	0.31	0.08	2.23	1.64	15.56	4.54	25.33	5.63
**17.10**	140.49	50.95	370.21	117.61	28.94	14.36	889.10	450.86	220.22	127.59	43.93	37.10	8.41	3.36	24.77	10.33	0.45	0.15	1.96	1.04	22.07	10.00	25.80	9.44
**CTRL**	187.00	76.45	320.47	161.10	24.93	11.50	3782.47	1646.88	761.72	490.96	135.32	53.45	13.99	7.82	39.17	25.26	0.34	0.09	1.85	0.62	32.53	18.26	31.56	14.48

Mean values of 3 biological and 2 technical replicates ± SD are shown.
