# Supplementary material for: Modification of Barley Plant Productivity Through Regulation of Cytokinin Content by Reverse-Genetics Approaches
Source: Front Plant Sci. 2018 Nov 27;9:1676. doi: 10.3389/fpls.2018.01676 (PMC6277847; doi:10.3389/fpls.2018.01676)
Supplement: Supplementary file 7 [file Table_7.pdf]

Table S7. Cytokinin content in KD-CKX1 (5.8; 4.3; 17.10; 21.4) and KO-CKX1 mutant lines (48.1; 39.4; 40.4) of stages 49, 59 and 71.

Stage 49 (booting: first awns visible (in awned forms only))																								
	**tZ**		**tZR**		**tZ9G**		**tZMP**		**cZ**		**cZR**		**cZMP**		**DHZR**		**iP**		**IPR**		**iPMP**		**iP9G**	
**Line**	**Mean**	**SD**	**Mean**	**SD**	**Mean**	**SD**	**Mean**	**SD**	**Mean**	**SD**	**Mean**	**SD**	**Mean**	**SD**	**Mean**	**SD**	**Mean**	**SD**	**Mean**	**SD**	**Mean**	**SD**	**Mean**	**SD**
**48.1**	14.21	8.12	10.12	3.53	4.36	1.94	20.25	5.21	28.44	2.49	97.40	33.02	52.08	14.04	UD	UD	12.69	4.44	18.80	6.21	8.23	4.43	4.70	2.33
**39.4**	27.06	13.27	40.43	15.81	17.00	3.51	45.96	17.14	47.67	4.91	104.46	18.52	53.95	6.74	UD	UD	11.74	5.14	26.92	5.10	13.16	2.83	4.12	0.91
**40.4**	55.86	27.63	25.81	12.21	10.71	3.62	62.59	18.51	39.78	1.96	146.54	59.49	53.25	7.36	UD	UD	17.39	7.78	33.71	10.51	22.36	12.59	8.30	3.75
**CTRL1**	30.22	20.33	21.11	16.43	6.57	1.97	28.82	9.44	44.33	23.08	91.23	24.09	52.06	12.66	UD	UD	10.39	4.99	20.23	8.14	9.90	6.75	2.63	0.92
**CTRL2**	29.76	12.71	38.89	11.73	16.80	3.18	49.85	11.31	35.60	3.56	79.93	11.72	40.02	6.83	UD	UD	6.98	1.89	27.26	3.62	10.17	1.96	3.63	0.19
**CTRL3**	25.97	18.69	22.78	5.75	14.63	4.43	23.68	6.00	42.90	8.71	138.23	68.38	51.80	10.23	UD	UD	8.29	3.72	25.86	6.25	14.68	2.83	5.10	1.27
**5.8**	43.08	16.73	28.96	15.91	13.45	3.36	49.15	7.57	38.40	3.60	125.50	20.48	55.57	7.31	UD	UD	9.81	4.45	30.98	6.62	15.79	6.03	4.73	1.14
**4.3**	32.82	21.13	37.66	15.99	13.17	4.12	68.98	23.30	39.40	1.94	152.40	54.71	58.81	15.11	UD	UD	7.72	4.31	30.56	5.61	13.84	2.69	2.43	1.11
**17.10**	26.53	6.77	37.22	9.31	21.98	6.61	89.08	47.02	48.01	7.22	137.25	38.93	49.01	7.24	UD	UD	5.11	0.79	31.20	5.38	10.74	2.57	3.83	0.81
**21.4**	14.44	7.43	15.17	7.05	5.79	1.56	33.99	10.67	41.50	25.49	140.89	68.12	56.09	21.87	UD	UD	10.77	4.90	30.12	15.85	13.77	5.27	7.60	4.37
**Stage 59 (end of heading: inflorescence fully emerged)**																								
	**tZ**		**tZR**		**tZ9G**		**tZMP**		**cZ**		**cZR**		**cZMP**		**DHZR**		**iP**		**IPR**		**iPMP**		**iP9G**	
	**Mean**	**SD**	**Mean**	**SD**	**Mean**	**SD**	**Mean**	**SD**	**Mean**	**SD**	**Mean**	**SD**	**Mean**	**SD**	**Mean**	**SD**	**Mean**	**SD**	**Mean**	**SD**	**Mean**	**SD**	**Mean**	**SD**
**48.1**	26.45	13.43	38.54	17.93	5.92	1.75	42.78	22.50	31.98	6.27	51.80	8.36	34.60	3.70	3.58	0.86	14.26	7.08	21.88	4.46	15.72	7.77	2.63	0.75
**39.4**	53.02	15.75	119.47	94.16	18.88	6.15	41.51	20.43	32.93	5.95	65.06	13.08	33.02	3.48	16.14	19.15	8.98	2.79	21.18	5.72	12.00	7.29	1.43	0.58
**40.4**	146.02	106.52	175.22	117.15	18.98	7.50	100.08	80.19	UD	UD	74.48	5.33	32.42	4.11	22.45	10.59	13.33	9.66	22.01	3.51	8.51	5.91	1.92	0.50
**CTRL1**	46.99	25.54	75.98	21.13	12.18	4.56	46.13	16.48	28.82	13.50	59.50	10.73	39.80	6.74	4.70	1.78	12.06	7.17	24.74	4.94	17.50	5.22	1.98	0.58
**CTRL2**	17.84	4.01	68.33	15.73	33.77	7.17	27.22	12.59	28.32	2.11	50.66	11.04	23.02	2.30	7.73	1.26	5.48	1.37	10.30	3.18	8.26	2.01	2.03	0.41
**CTRL3**	18.35	8.96	42.31	15.74	20.20	7.38	30.87	8.39	20.11	2.45	49.26	17.21	29.15	2.81	5.56	2.51	7.97	2.36	13.38	3.93	11.84	6.17	2.30	1.14
**5.8**	40.77	9.59	68.97	30.82	19.24	7.48	25.42	9.76	40.03	6.77	89.20	12.40	37.38	2.82	7.39	1.79	13.19	8.85	31.38	7.02	25.21	15.66	2.56	0.62
**4.3**	6.30	2.37	76.55	18.51	13.64	3.81	43.74	14.12	UD	UD	76.92	17.37	64.31	1.36	9.30	2.56	5.27	0.84	14.90	4.11	15.35	6.85	1.10	0.39
**17.10**	3.94	2.16	46.75	10.34	29.89	10.73	43.02	16.63	31.85	8.06	53.39	11.71	39.42	8.17	6.53	1.15	5.80	0.46	10.12	2.09	11.18	1.68	2.12	0.35
**21.4**	10.95	5.96	60.00	21.05	6.56	0.75	15.65	7.51	17.64	1.55	45.37	5.44	33.92	8.54	2.76	0.46	7.69	2.18	8.12	1.83	13.73	3.68	2.58	0.59

Stage 71 (watery ripe: first grains have reached half their final size)																								
	**tZ**		**tZR**		**tZ9G**		**tZMP**		**cZ**		**cZR**		**cZMP**		**DHZR**		**iP**		**IPR**		**iPMP**		**iP9G**	
	**Mean**	**SD**	**Mean**	**SD**	**Mean**	**SD**	**Mean**	**SD**	**Mean**	**SD**	**Mean**	**SD**	**Mean**	**SD**	**Mean**	**SD**	**Mean**	**SD**	**Mean**	**SD**	**Mean**	**SD**	**Mean**	**SD**
**48.1**	78.52	12.12	244.30	139.68	41.75	10.05	244.79	109.51	UD	UD	126.24	49.88	UD	UD	93.23	35.14	7.33	2.26	7.31	2.94	4.44	2.09	0.58	0.25
**39.4**	47.13	44.18	331.54	154.21	41.05	30.02	405.92	335.62	UD	UD	UD	UD	UD	UD	73.64	20.87	8.73	2.05	12.89	7.40	5.46	1.89	0.29	0.02
**40.4**	3042.40	332.50	246.49	32.5	43.25	12.30	299.57	36.80	UD	UD	UD	UD	UD	UD	102.92	35.20	11.32	4.80	17.26	4.80	4.01	1.50	0.14	0.01
**CTRL1**	27.46	19.78	345.73	59.80	21.54	3.60	37.09	9.23	UD	UD	167.8	18.3	30.67	5.4	23.69	6.02	39.30	16.97	26.73	8.26	4.27	1.67	0.59	0.20
**CTRL2**	ND	ND	206.44	78.52	51.02	6.70	56.83	21.66	UD	UD	100.33	11.80	UD	UD	18.98	5.46	24.65	0.39	16.79	7.84	3.77	1.19	0.25	0.02
**CTRL3**	45.05	18.83	458.52	143.78	UD	UD	153.62	113.63	UD	UD	UD	UD	20.60	3.80	39.05	17.82	36.18	11.41	29.56	8.02	4.58	1.37	0.28	0.12
**5.8**	1395.58	337.49	140.11	49.02	29.98	17.93	43.11	11.25	UD	UD	123.18	28.21	30.55	3.25	32.37	5.85	9.09	2.19	18.47	7.68	4.49	0.67	0.42	0.03
**4.3**	4349.69	456.50	134.40	54.50	52.38	6.70	76.31	8.50	UD	UD	UD	UD	UD	UD	56.80	15.60	34.02	3.20	13.46	8.90	3.78	1.30	UD	UD
**17.10**	12.35	2.27	173.08	77.45	30.91	5.80	32.60	4.68	UD	UD	76.36	8.40	UD	UD	28.29	2.34	33.48	2.68	17.06	6.45	4.35	2.16	0.70	0.32
**21.4**	4.55	1.44	30.83	23.36	51.15	21.75	23.08	7.85	UD	UD	95.75	15.06	30.55	11.46	16.52	3.12	5.77	2.55	9.32	2.36	4.20	1.30	0.39	0.15

Mean values of 8(5) biological replicates ± SD are shown. ND – not determined; UD – under detection limit
