# Supplementary material for: Modification of Barley Plant Productivity Through Regulation of Cytokinin Content by Reverse-Genetics Approaches
Source: Front Plant Sci. 2018 Nov 27;9:1676. doi: 10.3389/fpls.2018.01676 (PMC6277847; doi:10.3389/fpls.2018.01676)
Supplement: Supplementary file 10 [file Image_1.pdf]

GGATCCGGGTCCGGCACTTCGGCGGCAAGTGGAAGCACTTCGTGGAGATGAAGGACAAG
TACGACCCCAAGAAGCTGCTCTCCCCTGGCCAAGACATATTCAAACTGCTCCTTTGAGTA
GAGATAACAATTATACAGTATACTGTAGTTGTTACTACTAGTGGATTAACGTACGTGTGG
ACAGTAACACAGCAGTTTAACTTTAATTTCCCGTTTTTACACATGCAGTAGCAAATTTGAT
AGTAGGTGGCGATATC

Figure S1. Sequence of *HvCKX1* gene used for design of silencing cassette. Sequence consists of 5´ end of the ORF and 3’ untranslated region (3´UTR) of *HvCKX1* gene and it is bordered by *Bam*HI and *Eco*RI restriction sites (highlighted in yellow).
