# Supplementary material for: Modification of Barley Plant Productivity Through Regulation of Cytokinin Content by Reverse-Genetics Approaches
Source: Front Plant Sci. 2018 Nov 27;9:1676. doi: 10.3389/fpls.2018.01676 (PMC6277847; doi:10.3389/fpls.2018.01676)
Supplement: Supplementary file 11 [file Image_2.pdf]

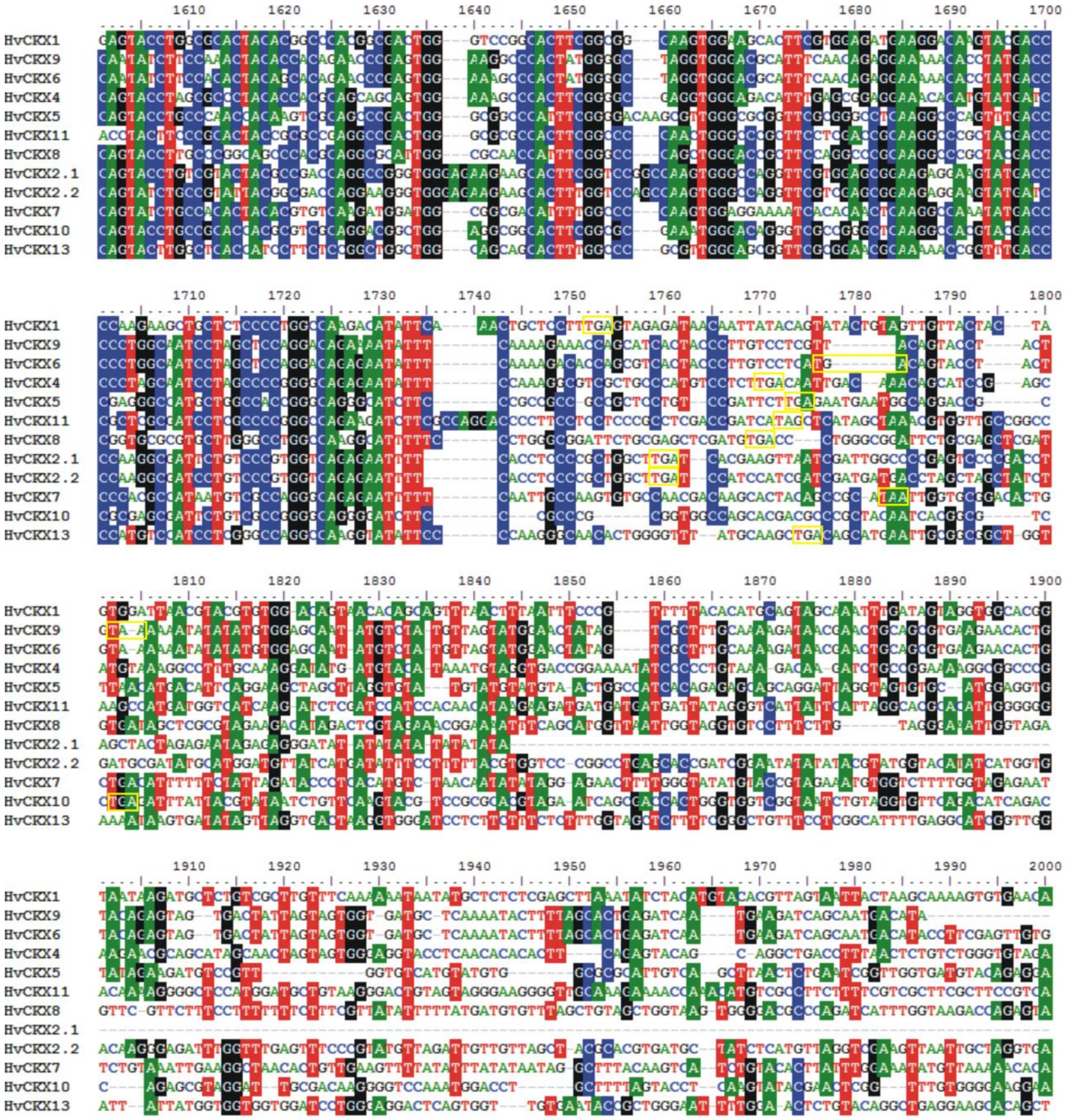

Figure S2. Sequence alignment of barley *CKX* genes. Alignment was performed by BioEdit Sequence Alignment Editor. Identity/similarity between individual sequences and *HvCKX1* silencing cassette are shading with colour table. Stop codons are labelled with yellow rectangle.
