# Supplementary material for: Modification of Barley Plant Productivity Through Regulation of Cytokinin Content by Reverse-Genetics Approaches
Source: Front Plant Sci. 2018 Nov 27;9:1676. doi: 10.3389/fpls.2018.01676 (PMC6277847; doi:10.3389/fpls.2018.01676)
Supplement: Supplementary file 12 [file Image_3.pdf]

A)

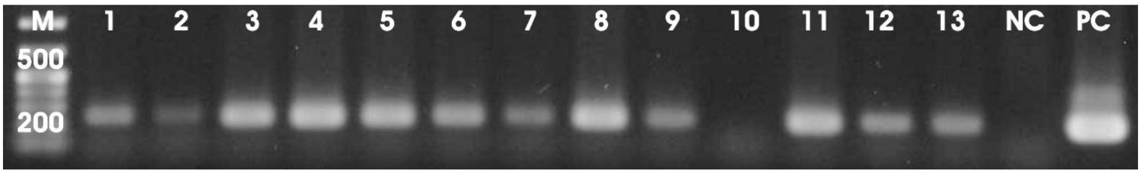

B)

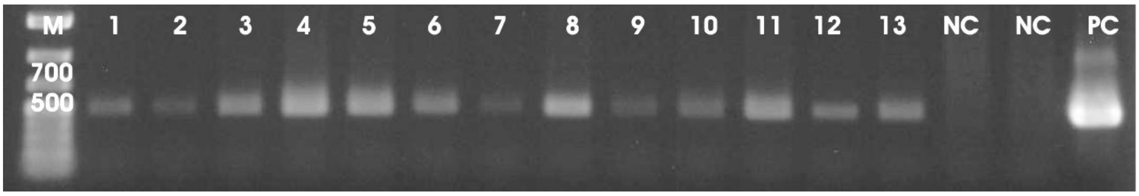

Figure S3. Example of PCR screening for the presence of sense (A) and antisense (B) components of *SC-HvCKX1* silencing cassette. Lines 1-13 contain products obtained by PCR amplification using genomic DNA as a template extracted from selected 13 plants from T0 generation. Five ng of plasmid pBract207::*SC_HvCK1* was used as a positive control (PC). DNA extracted from control plants, regenerated from non-transformed embryos was used as a negative control (NC). Five µL of GeneRuler™ 1 kb Plus DNA Ladder was used as marker (M).
