# Supplementary material for: Modification of Barley Plant Productivity Through Regulation of Cytokinin Content by Reverse-Genetics Approaches
Source: Front Plant Sci. 2018 Nov 27;9:1676. doi: 10.3389/fpls.2018.01676 (PMC6277847; doi:10.3389/fpls.2018.01676)
Supplement: Supplementary file 13 [file Image_4.pdf]

A)

Query	Subject	Score	Identities (Query length)	Percentage	Expect
Query1	HORVU3Hr1G019850|chr3H:58407698-58410314|HC_G|cytokinin	37.4	20/20 (20)	100	0.004
Query1	HORVU0Hr1G013800|chrUn:77795619-77830417|HC_G|oligopeptide	31.9	17/17 (20)	100	0.17
Query1	HORVU5Hr1G095440|chr5H:598313214-598325798|HC_G|tornado	31.9	17/17 (20)	100	0.17
Query1	HORVU5Hr1G095460|chr5H:598320669-598325900|HC_G|oligopeptide	31.9	17/17 (20)	100	0.17
Query1	HORVU5Hr1G086250|chr5H:576150954-576183325|HC_G|SAUR-like	28.3	15/15 (20)	100	2.1
Query1	HORVU3Hr1G046180|chr3H:306942032-306970001|HC_G|Protein	28.3	15/15 (20)	100	2.1
Query1	HORVU4Hr1G059060|chr4H:493802120-493805262|HC_G|ADP-ribosylation	28.3	15/15 (20)	100	2.1
Query1	HORVU5Hr1G086240|chr5H:576148495-576183422|HC_G|SAUR-like	28.3	15/15 (20)	100	2.1
Query1	HORVU1Hr1G067230|chr1H:477443466-477449042|HC_G|metacaspase	28.3	17/18 (20)	94	2.1
Query1	HORVU5Hr1G066370|chr5H:506740106-506766525|HC_G|Copper-transporting	28.3	15/15 (20)	100	2.1

B)

```
                         PAM  target sequence
HORVU3Hr1G019850         CCGCGGCGTCTCCTACGGCGCAC    on-target
HORVU0Hr1G013800         CTGCGGCGTCTCCTACGGCGGCG    no NGG PAM
HORVU5Hr1G095440         CTGCGGCGTCTCCTACGGCGGCG    no NGG PAM
HORVU5Hr1G095460         CTGCGGCGTCTCCTACGGCGGCG    no NGG PAM
HORVU5Hr1G086250         CGACCGCGTCTCCTACGGCGAGC    no NGG PAM
HORVU3Hr1G046180         CGACCGCGTCTCCTACGGCGAGC    no NGG PAM
HORVU4Hr1G059060         GGCCAGCGTCTCCTACGGCGTCT    no NGG PAM
HORVU5Hr1G086240         CGACCGCGTCTCCTACGGCGAGC    no NGG PAM
HORVU1Hr1G067230         CAGCGGCGTGTCCTACGGCGCGC    no NGG PAM
HORVU5Hr1G066370         CGACCGCGTCTCCTACGGCGAGC    no NGG PAM
```

Figure S4. Off-target analysis for *HvCKX1* guide sequence. (A) Table representing 10 best results of Blast search against barley genome sequence (http://webblast.ipk-gatersleben.de/barley_ibsc/viroblast.php; assembly_WGSMorex) and **(B)** sequence alignments of the respective regions covering guide RNA target sequence including PAM
