# Supplementary material for: Modification of Barley Plant Productivity Through Regulation of Cytokinin Content by Reverse-Genetics Approaches
Source: Front Plant Sci. 2018 Nov 27;9:1676. doi: 10.3389/fpls.2018.01676 (PMC6277847; doi:10.3389/fpls.2018.01676)
Supplement: Supplementary file 14 [file Image_5.pdf]

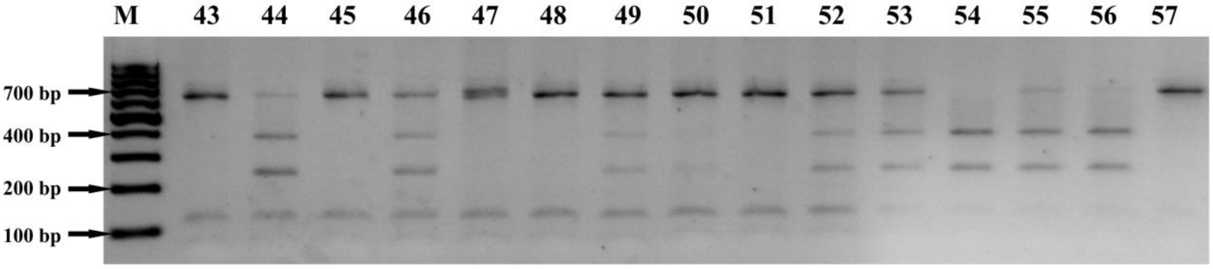


**Figure S5. Restriction analysis of *HvCKX1* PCR product from several KO-CKX1 mutants. A** 756 bp PCR product of the first *HvCKX1* exon was restricted with *Bsa*HI. Amplicons with mutations on target site gave the restriction products of 582, 114 and 59 bp and non-mutated amplicons products of 355, 228, 114 and 59 bp. Five µl of GeneRuler™ 100 bps DNA Ladder was used as marker (M); the numbers represents the designation of the KO-CKX1 mutants.
